# Targeting KPNB1 overcomes TRAIL resistance by regulating DR5, Mcl-1 and FLIP in glioblastoma cells

**DOI:** 10.1038/s41419-019-1383-x

**Published:** 2019-02-11

**Authors:** Zhi-Chuan Zhu, Ji-Wei Liu, Can Yang, Ming-Jie Li, Rong-Jie Wu, Zhi-Qi Xiong

**Affiliations:** 1 State Key Laboratory of Neuroscience, Institute of Neuroscience, CAS Center for Excellence in Brain Science and Intelligence Technology, Chinese Academy of Sciences, Shanghai, China; 2 Shanghai Mental Health Center, Shanghai Jiao Tong University School of Medicine, Shanghai, China; 3 University of Chinese Academy of Sciences, Beijing, China; 4 School of Life Science and Technology, ShanghaiTech University, Shanghai, China

## Abstract

Tumor necrosis factor-related apoptosis-inducing ligand (TRAIL) is a cytokine with potential anticancer effect, but innate and adaptive TRAIL resistance in majority of cancers limit its clinical application. Karyopherin β1 (KPNB1) inhibition in cancer cells has been reported to abrogate the nuclear import of TRAIL receptor DR5 and facilitate its localization on the cell surface ready for TRAIL stimulation. However, our study reveals a more complicated mechanism. Genetic or pharmacological inhibition of KPNB1 potentiated TRAIL-induced apoptosis selectively in glioblastoma cells mainly by unfolded protein response (UPR). First, it augmented ATF4-mediated DR5 expression and promoted the assembly of death-inducing signaling complex (DISC). Second, it freed Bax and Bak from Mcl-1. Third, it downregulated FLIP_L_ and FLIP_S_, inhibitors of caspase-8 cleavage, partly through upregulating ATF4–induced 4E-BP1 expression and disrupting the cap-dependent translation initiation. Meanwhile, KPNB1 inhibition-induced undesirable autophagy and accelerated cleaved caspase-8 clearance. Inhibition of autophagic flux maintained cleaved caspase-8 and aggravated apoptosis induced by KPNB1 inhibitor plus TRAIL, which were abolished by caspase-8 inhibitor. These results unveil new molecular mechanism for optimizing TRAIL-directed therapeutic efficacy against cancer.

## Introduction

Tumor necrosis factor-related apoptosis-inducing ligand (TRAIL) belongs to the tumor necrosis factor superfamily of cytokines and is involved in inflammation and immunosurveillance. It is expressed in both normal and tumor cells. TRAIL induces apoptosis by engaging its functional receptors DR4 (TRAIL-R1) and DR5 (TRAIL-R2). Upon TRAIL stimulation, TRAIL receptors undergo homotrimerization and recruit Fas-associated protein with death domain (FADD). FADD turns to recruit caspase-8. Assembly of this death-inducing signaling complex (DISC) promotes caspase-8 processing and activation. In certain types of cells, cleaved caspase-8 directly cleaves effector caspases like caspase-3 to induce apoptosis, while in other cells the intrinsic mitochondrial apoptotic signaling amplifies the death signal. In the latter case, Bid, truncated by cleaved caspase-8, translocates to the mitochondria and binds pro-survival Bcl-2 proteins like Bcl-xL or pro-apoptotic Bcl-2 proteins like Bax and Bak to facilitate mitochondria outer membrane permeabilization (MOMP). This leads to the release of cytochrome c and other pro-apoptotic factors into the cytosol, the activation of effector caspases and the induction of apoptosis^1,2^.

Clinical trials revealed the safety but disappointed clinical benefits of TRAIL-based therapies^2,3^. Multiple factors in TRAIL receptor signaling determine TRAIL responsiveness, including the expression, localization, and clustering of TRAIL receptors, the assembly and distribution of DISC and the expression of Bcl-2 family proteins and inhibitors of apoptosis proteins^1,4^. Therapeutic strategies modulating these factors to improve TRAIL response are urgently needed.

Karyopherin β1 (KPNB1) participates in the nuclear import of many cancer-associated proteins including DR5^5–8^. KPNB1 transports DR5 into the nucleus, while knocking down KPNB1 restores DR5 protein level on the cell surface and TRAIL sensitivity of cancer cells^8^. We demonstrated previously that KPNB1 inhibition perturbed proteostasis and activated PERK signaling branch of unfolded protein response (UPR) in glioblastoma cells^9^. Given that PERK branch regulates the expression of DR5 and other determinants of TRAIL susceptibility^10,11^, we envisage that KPNB1 inhibition may overcome TRAIL resistance via UPR rather than simply abolishing DR5 nuclear import. In the present study, we show that KPNB1 inhibition results in DR5 upregulation, Mcl-1 disability and FLIP downregulation via UPR. Combination of KPNB1 inhibitor and TRAIL along with the lysosome inhibitor uncoupling pro-survival autophagy has potential in cancer treatment.

## Results

### Inhibition of KPNB1 sensitizes glioblastoma cells to TRAIL-induced apoptosis

**Fig. 1:**
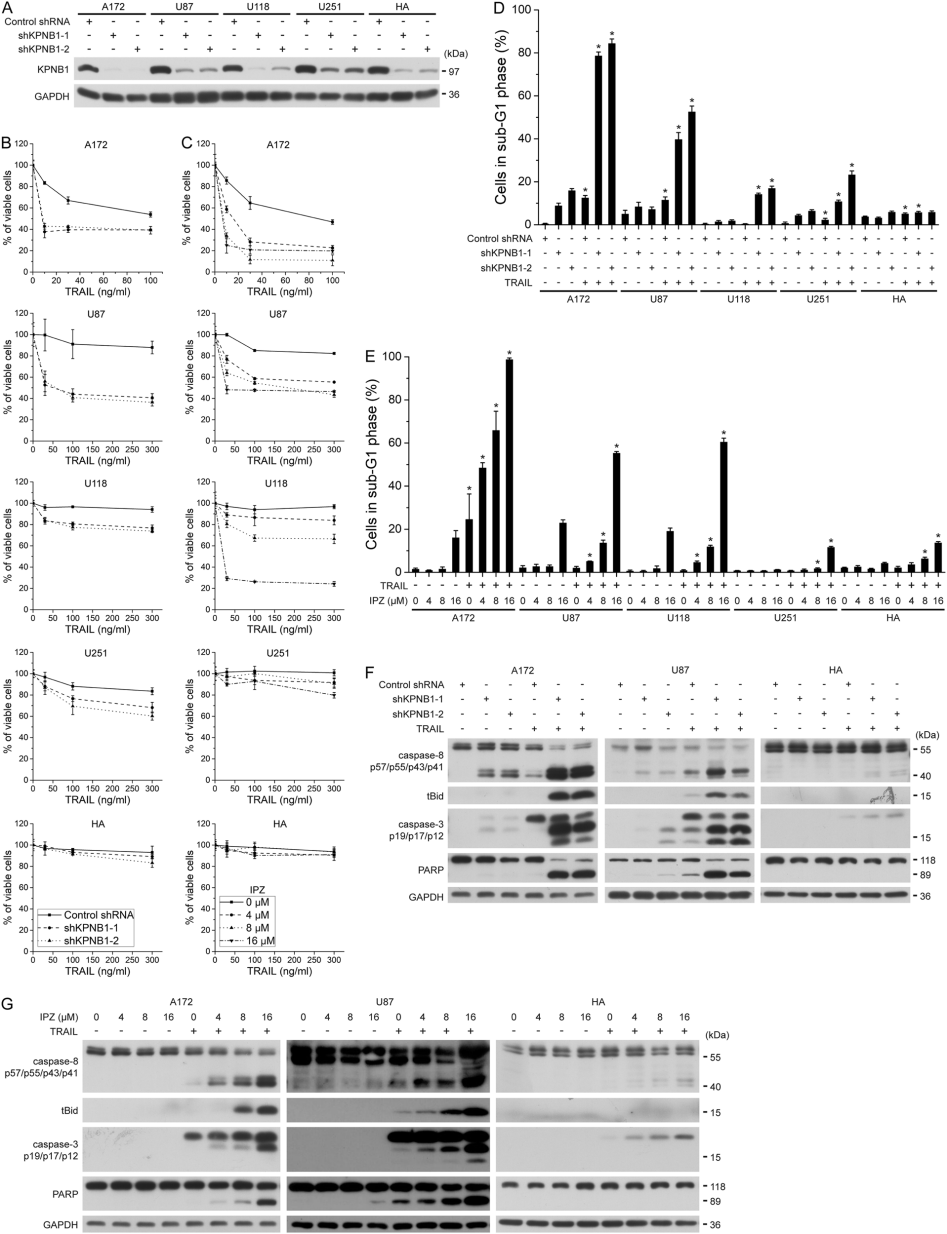
Inhibition of KPNB1 sensitizes glioblastoma cells to TRAIL-induced apoptosis. **a** A172, U87, U118, U251, and HA cells were infected lentiviruses encoding shKPNB1s and a scrambled shRNA (Control shRNA). Knockdown efficacy of shRNAs was validated by western blot. Molecular weight of proteins is indicated at the right-hand side. **b**, **c** Cells either expressing shKPNB1s (**b**) or pretreated with indicated dose of IPZ for 24 h (**c**) were treated with indicated dose of human recombinant TRAIL for 24 h. Cell viability was measured by MTT assay. Results represent the mean ± SD from one of the three independent experiments in triplicates. **d**, **e** Cells pretreated as indicated were treated with TRAIL (30 ng/ml) for 24 h. The percentage of apoptotic cells was analyzed by flow cytometry. Results represent mean ± SD from three independent experiments. *P < 0.05 compared with the corresponding group without TRAIL treatment. **f**, **g** U87 cells pretreated as indicated were further treated with TRAIL (30 ng/ml) for 6 h. Proteins in the death receptor signaling were analyzed by western blot. GAPDH was used as the loading control

It was reported that KPNB1 knockdown primed cancer cells to TRAIL-induced apoptosis by upregulating cell surface DR5^8^. Consistently, in our study, KPNB1 shRNAs (shKPNB1–1, 2) or specific inhibitor importazole (IPZ) potentiated TRAIL cytotoxicity in A172, U87, U118, U251 human glioblastoma cells but not in human fetal astrocytes (HA) (Fig. 1a–c). In A172 and U87 cells, KPNB1 inhibition plus TRAIL-induced robust cell death and activation of the death receptor apoptotic signaling in terms of the cleavage of caspase-8 (p43/p41), Bid, caspase-3 (intermediate p19 and effector p17/p12) and PARP (Fig. 1d–g). Such effects were weaker in U251, U118 cells (Fig. 1d, e) and were weakest in HA cells (Fig. 1d–g). These results suggest that KPNB1 inhibition synergizes with TRAIL to selectively induce apoptosis in glioblastoma cells.

### KPNB1 inhibition increases total and cell surface DR5 level in glioblastoma cells

**Fig. 2:**
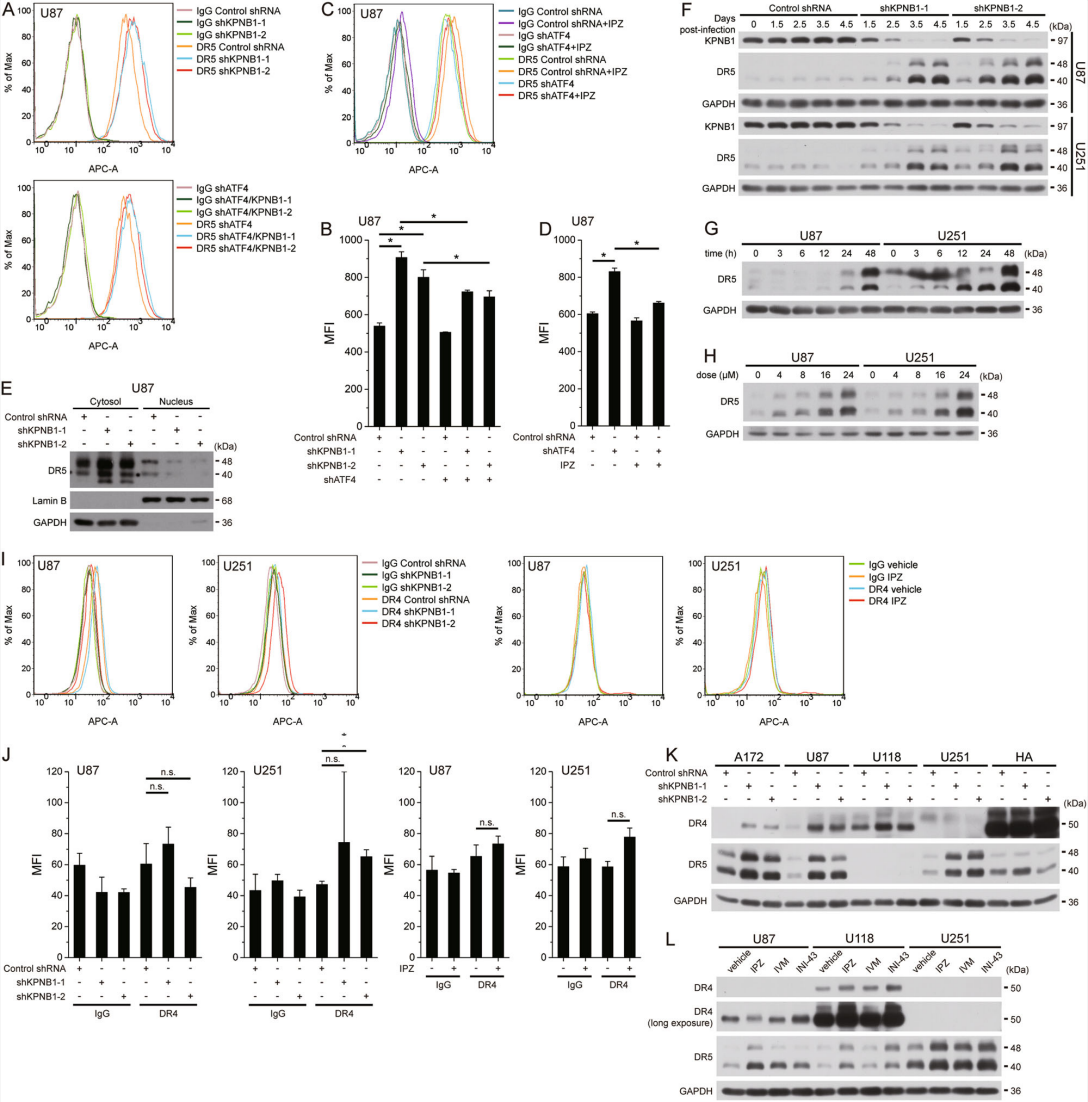
KPNB1 inhibition increases total and cell surface DR5 level in glioblastoma cells. **a**–**d** Intact shATF4-expressing U87 cells either expressing shKPNB1s (**a**) and (**b**) or treated with IPZ (16 μM) for 24 h (**c**) and **d** were stained with APC anti-human DR5 antibody or IgG isotype ctrl antibody. Cell surface DR5 levels were measure by flow cytrometry **a** and **c**. Mean fluorescence intensity (MFI) was shown in **b** and **d**. Results represent mean ± SD from three independent experiments. *P < 0.05. **e** Western blots analysis of cytosolic and nuclear DR5 level in shKPNB1-expressing U87 cells. Equal amount of cytosolic and nuclear protein was loaded. **f**–**h** U87 and U251 cells were harvested after shKPNB1-encoding lentiviruses infection (**f**) or IPZ (16 μM) treatment (**g**) for indicated times or after treating with various concentration of IPZ for 24 h (**h**) Levels of proteins were analyzed by western blot. **i**, **j** Intact U87 and U251 cells either expressing shKPNB1s or treated with IPZ (16 μM) for 24 h were stained with APC anti-human DR4 antibody or IgG isotype ctrl antibody. Cell surface DR4 levels were measured by flow cytometry (**i**). MFI was shown in (**j**). Results represent mean ± SD from three independent experiments. *P < 0.05. N.S. not significant. **k** Western blot analysis of DR4 and DR5 in shKPNB1-expressing A172, U87, U118, U251 and HA cells. **l** Western blot analysis of DR4 and DR5 in U87, U118 and U251 cells treated with IPZ (16 μM), IVM (16 μM) or INI-43 (8 μM) for 24 h. GAPDH was used as the loading control of total cell lysates or cytosolic fraction. Lamin B was used as the loading control of nuclear fraction

In consistent with previous findings^8^, both KPNB1 knockdown and IPZ treatment increased cell surface DR5 levels in U87 cells (Fig. 2a–d). Besides, KPNB1 knockdown attenuated DR5 nuclear import in U87 cells (Fig. 2e). Inconsistently, however, KPNB1 inhibition also increased total DR5 levels in U87 and U251 cells in a time- and dose-dependent manner (Fig. 2f–h). Meanwhile, KPNB1 knockdown and IPZ treatment did not generally increase cell surface DR4 level in U87 and U251 cells (Fig. 2i, j). DR4 mean fluorescence intensity (MFI) was comparable to that of isotype IgG in U87 and U251 cells and lower than DR5 MFI in U87 cells (Fig. 2a–d, i, j), recapitulating previous result^12^ and indicating low cell surface expression of DR4 in these cells. KPNB1 knockdown upregulated total DR4 and DR5 in all tested glioblastoma cell lines (Fig. 2k). Each KPNB1 inhibitor (IPZ, IVM, and INI-43) upregulated DR5 in U87, U118, and U251 cells. However, IPZ upregulated DR4 only in U118 cells and INI-43 upregulated DR4 only in U87 and U118 cells (Fig. 2l). U118 cells also had high DR5 and low DR4 on the cell surface despite expressing relatively high total DR4 and low total DR5 (Fig. 2k, l and Supplementary Fig. S1A, B). IPZ but not KPNB1 knockdown increased cell surface DR4 and DR5 in U118 cells (Supplementary Fig. S1A, B), possibly explaining lower TRAIL response in shKPNB1 cells than that in IPZ-treated cells (Fig. 1d, e). These results suggest that KPNB1 inhibition upregulates cell surface DR5 level by inducing DR5 expression in addition to suppressing DR5 internalization, while it does not uniformly increase cell surface DR4 level albeit affecting total DR4 level in some cases.

### KPNB1 inhibition enhances TRAIL sensitivity by promoting ATF4-mediated DR5 expression

**Fig. 3:**
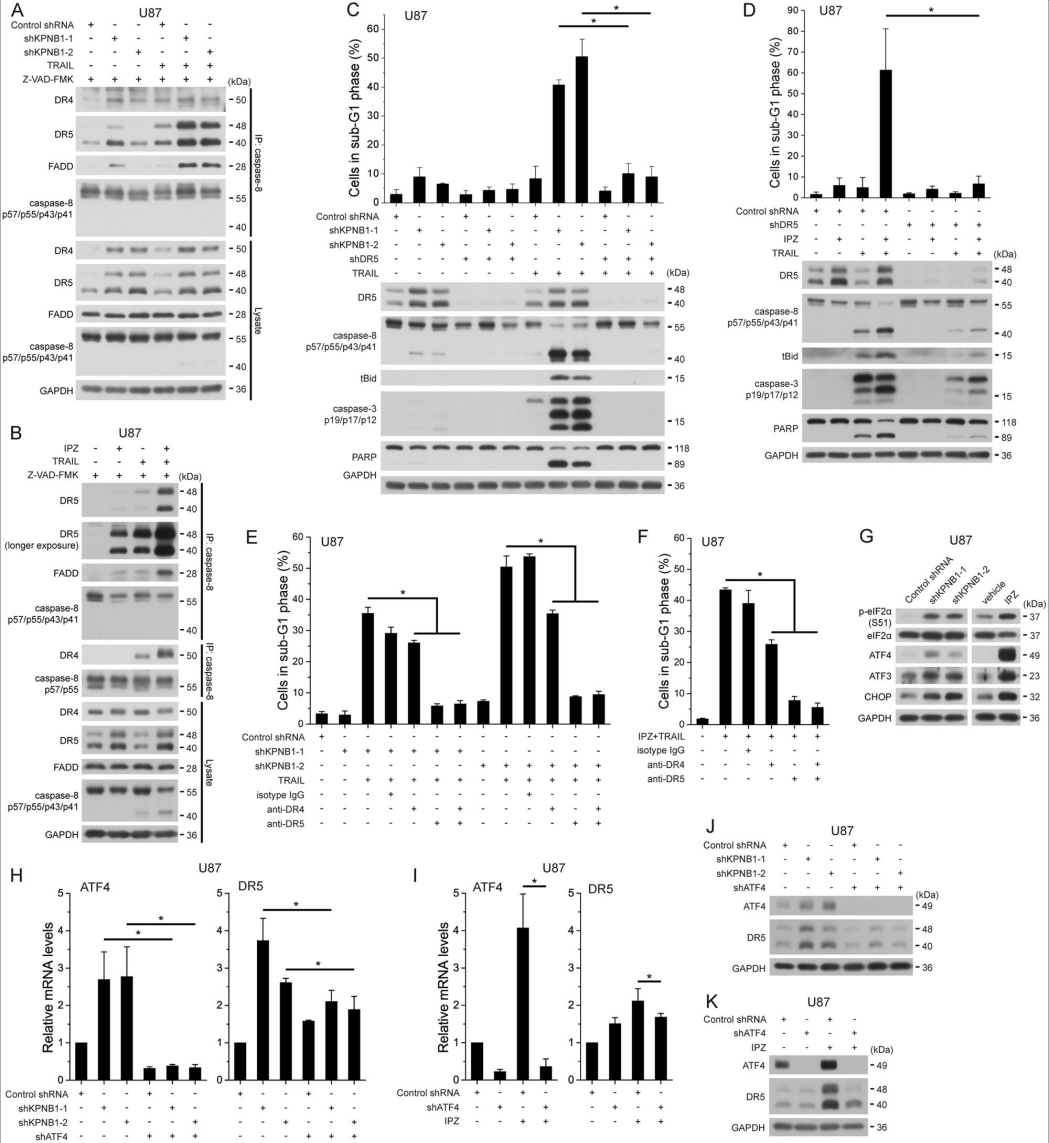
KPNB1 inhibition enhances TRAIL sensitivity by promoting ATF4-mediated DR5 expression. **a**, **b** shKPNB1-expressing (**a**) or IPZ (16 μM)-treated (**b**) U87 cells were treated with pan-caspase inhibitor Z-VAD-FMK (20 μM) for 5 h to block caspase-8 cleavage and further treated with TRAIL (100 ng/ml) for 1 h. DISC in cell lysates was immunoprecipitated using caspase-8 antibody followed by western blot. **c** U87 cells expressing shKPNB1s and/or shDR5 were treated with TRAIL (50 ng/ml, 6 h, for western blot; 30 ng/ml, 24 h, for flow cytometry, the same below unless noted) and subjected to western blot or flow cytometry. Results represent mean ± SD from three independent experiments. *P < 0.05. **d** U87 cells expressing shDR5 were treated with IPZ (16 μM) for 24 h and further treated with TRAIL, then subjected to western blot or flow cytometry. Results represent mean ± SD from four independent experiments. *P < 0.05. **e**, **f** U87 cells either expressing shKPNB1s (**e**) or pretreated with IPZ (16 μM) for 24 h (**f**) were treated with antagonistic antibody to DR4 or DR5 or IgG1 isotype control antibody (all 5 μl/ml) for 1 h and further with TRAIL for 24 h, then subjected to flow cytometry. Results represent mean ± SD from three independent experiments. *P < 0.05. **g** Western blot analysis of indicated proteins in shKPNB1-expressing or IPZ (16 μM)-treated U87 cells. **h**, **i** Real-time PCR analysis of indicated genes in U87 cells expressing shKPNB1s and/or shATF4 (**h**), and in U87 cells expressing shATF4 and/or treated with IPZ (16 μM) **i** Results represent mean ± SD from three independent experiments. *P < 0.05. **j**, **k** Western blot analysis of indicated proteins in U87 cells treated as in **h** and **i**. GAPDH was used as the loading control

In U87 cells, IPZ dose-dependent DR5 upregulation correlates with the synergism of IPZ and TRAIL (Fig. 1c, e and g). KPNB1 knockdown or IPZ treatment strongly promoted TRAIL-induced assembly of DISC core members caspase-8, FADD, and DR5 but not DR4 (Fig. 3a, b). Apoptosis induced by KPNB1 inhibition and TRAIL treatment was reversed by DR5 knockdown in U87 cells (Fig. 3c, d). Antagonistic antibody to DR5 neutralizing cell surface DR5^13^ also reversed such apoptosis in U87 cells, whereas the effect of antagonistic antibody to cell surface DR4 was weaker (Fig. 3e, f). Similar result was obtained in IPZ-treated U118 cells with upregulated cell surface DR4 and DR5 (Supplementary Fig. S1C). These results suggest that KPNB1 inhibition-upregulated cell surface DR5 mainly amplifies the death receptor signaling transduction favoring TRAIL-triggered apoptosis in tested glioblastoma cells.

ER stress regulators, p53, and NF-κB regulate DR5 transcription^10,14^. KPNB1 inhibition activated PERK signaling branch of UPR in U87 cells (Fig. 3g). Knocking down ATF4 reversed the upregulation of mRNA, total protein and cell surface DR5 upon KPNB1 inhibition (Fig. 2a–d and Fig. 3h–k). Knocking down CHOP that commonly enhances DR5 transcription under ER stress^15,16^ had a weaker effect (Supplementary Fig. S2A-D). KPNB1 inhibition also slowed DR5 degradation and downregulated E3 ligase c-Cbl which accounts for DR5 degradation and early phase TRAIL resistance^17^ (Supplementary Fig. S3A, B). Overexpression of c-Cbl reversed DR5 level mildly that was insufficient to attenuate apoptosis upon KPNB1 inhibition and TRAIL stimulation (Supplementary Fig. S3C, D). These results suggest that KPNB1 inhibition-upregulated DR5, mainly through ATF4, enhances TRAIL sensitivity in glioblastoma cells.

### KPNB1 inhibition compromises Mcl-1 to enhance TRAIL sensitivity

**Fig. 4:**
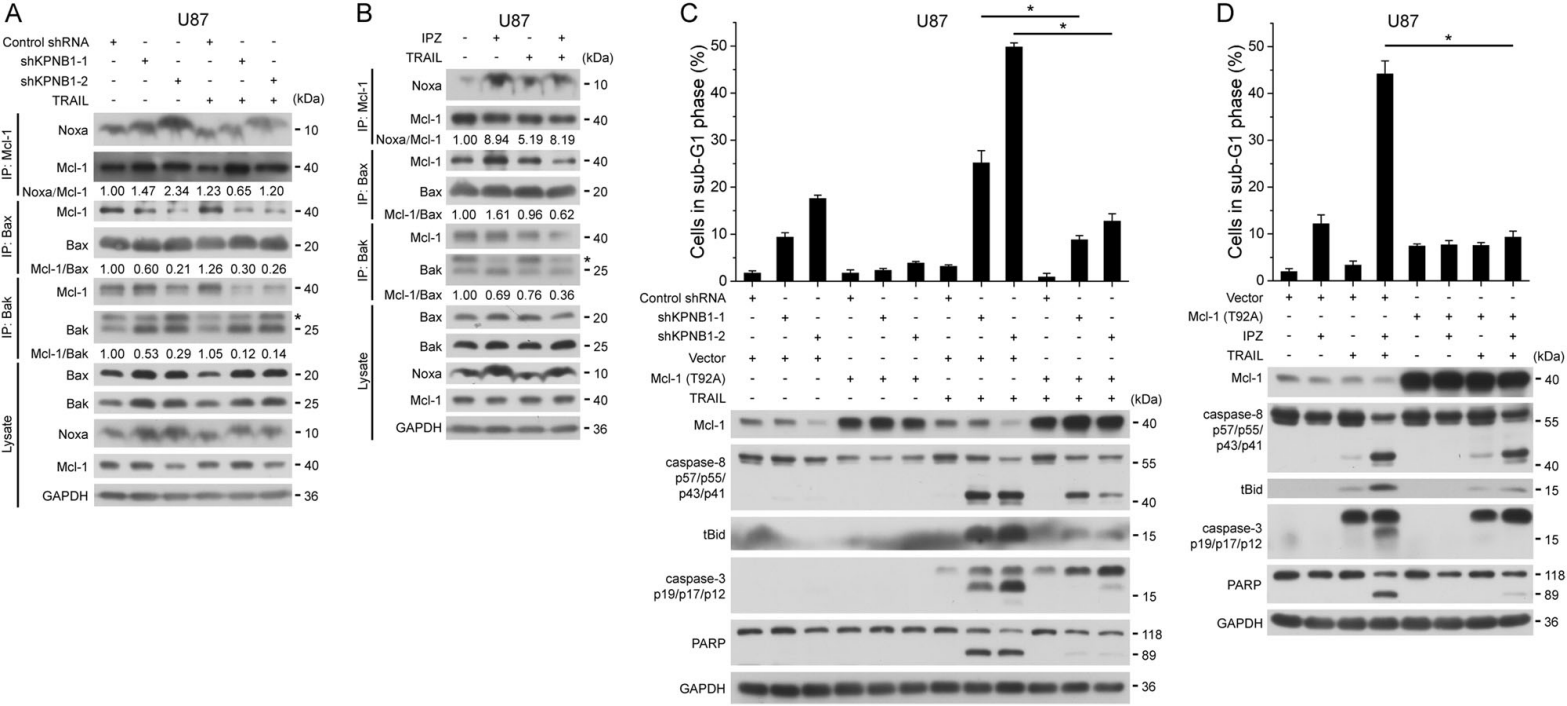
KPNB1 inhibition cripples Mcl-1 to potentiate TRAIL-induced apoptosis. **a**, **b** U87 cells either expressing shKPNB1s (**a**) or pretreated with IPZ (16 μM) for 24 h (**b**) were treated with TRAIL. Mcl-1, Bax and Bak in cell lysates was immunoprecipitated followed by western blot. *indicates IgG light chain. Indicated grayscale ratios were listed. **c** U87 cells expressing shKPNB1s and/or Mcl-1 (T92A) mutant were treated with TRAIL and subjected to western blot or flow cytometry. **d** U87 cells expressing Mcl-1 (T92A) mutant were treated with IPZ (16 μM) for 24 h and further with TRAIL, then subjected to flow cytometry and western blot. GAPDH was used as the loading control. Results represent mean ± SD from three independent experiments. *P < 0.05

Mcl-1 prevents MOMP when TRAIL-induced death receptor signaling turns to activate the mitochondrial apoptotic signaling^2^. In U87 cells, KPNB1 knockdown or IPZ treatment decreased binding of Mcl-1 to Bax and Bak (Fig. 4a, b). KPNB1 inhibition-upregulated Noxa, a transcriptional target of ATF4 and a Mcl-1 antagonizer^18^, and its binding to Mcl-1 (Fig. 4a, b). However, Noxa knockdown did not rescue apoptosis induced by IPZ and TRAIL (Supplementary Fig. S4). Overexpression of the non-degradable Mcl-1 (T92A) mutant partly reversed KPNB1 inhibition and TRAIL-induced apoptosis in U87 cells (Fig. 4c, d). These results suggest that KPNB1 inhibition compromises the anti-apoptotic function of Mcl-1 to foster TRAIL-induced apoptosis.

### KPNB1 inhibition cripples FLIP translation and TRAIL tolerance by ATF4/4E-BP1 axis

**Fig. 5:**
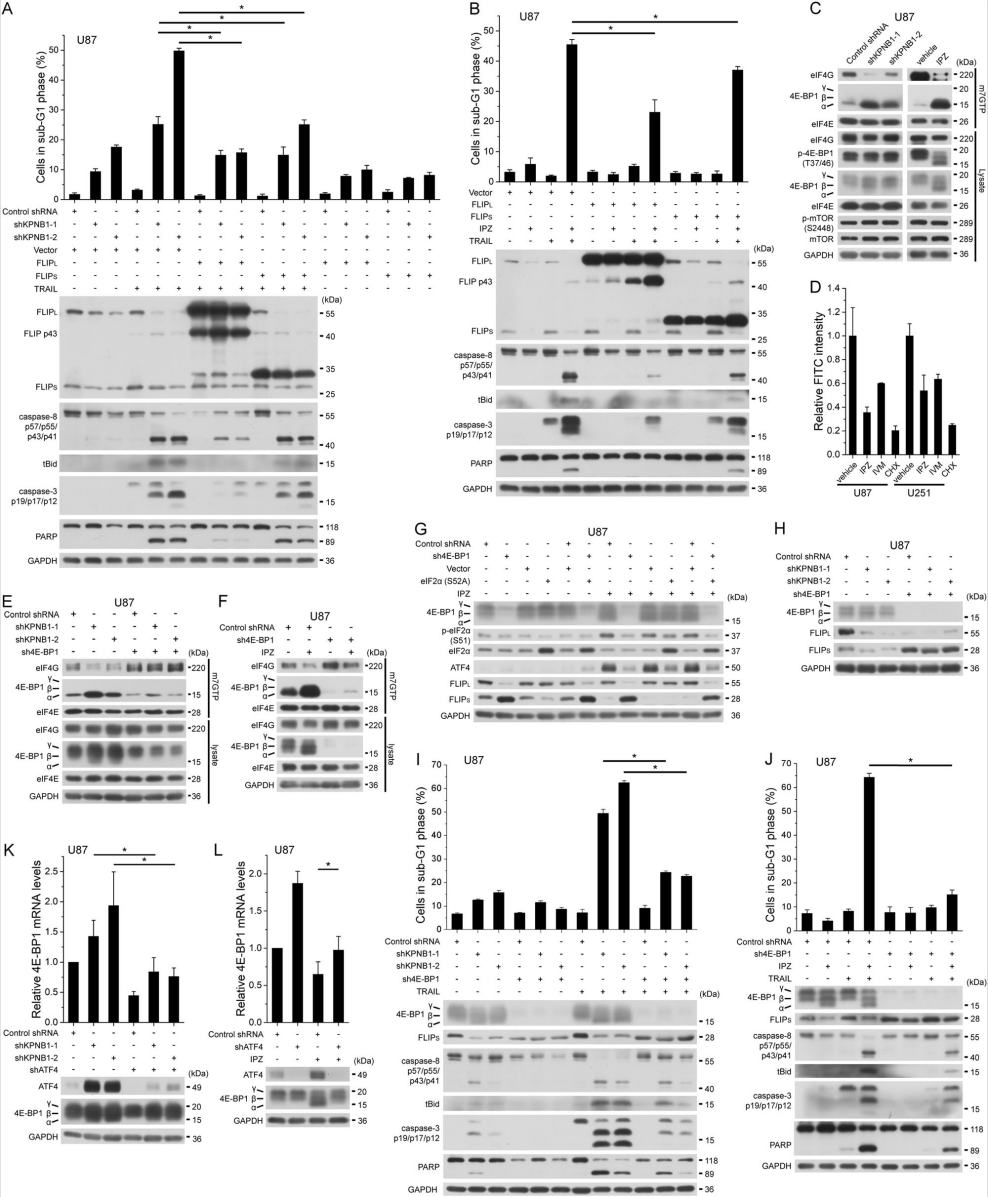
KPNB1 inhibition cripples cap-dependent translation of FLIP and TRAIL tolerance by ATF4/4E-BP1 axis. **a** U87 cells expressing shKPNB1s and FLIP_L_ or FLIP_S_ were treated with TRAIL and subjected to western blot or flow cytometry. Results represent mean ± SD from three independent experiments. **b** U87 cells expressing FLIP_L_ or FLIP_S_ were treated with IPZ (16 μM) for 24 h and further with TRAIL, then subjected to western blot and flow cytometry. Results represent mean ± SD from three independent experiments. **c** U87 cells expressing shKPNB1s or treated with IPZ (16 μM) for 24 h were subjected to cap-binding assay. **d** Protein synthesis was measured by OPP incorporation after treating U87 and U251 cells with 16 μM IPZ or IVM for 24 h, or with 100 μg/ml CHX for 4 h as a negative control for protein synthesis Results represent mean ± SD from one of the three independent experiments in triplicates. **e** U87 cells expressing shKPNB1s and/or sh4E-BP1 were subjected to cap-binding assay. **f** U87 cells expressing sh4E-BP1 were treated with IPZ (16 μM) for 24 h and subjected to cap-binding assay. **g** U87 cells expressing sh4E-BP1 and/or eIF2α (S52A) were treated with IPZ (16 μM) for 24 h and subjected to western blot. **h** U87 cells from **e** were subjected to western blot. **i** U87 cells expressing sh4E-BP1 were treated as in **a** and subjected to western blot and flow cytometry. Results represent mean ± SD from three independent experiments. **j** U87 cells expressing shKPNB1s and/or sh4E-BP1 were treated as in **b** and subjected to western blot or flow cytometry. Results represent mean ± SD from three independent experiments. **k** U87 cells expressing shKPNB1s and/or shATF4 were subjected to real-time PCR and western blot. Results represent mean ± SD from three independent experiments. **l** U87 cells expressing shATF4 were treated with IPZ (16 μM) for 24 h and subjected to real-time PCR and western blot. Results represent mean ± SD from three independent experiments. GAPDH was used as the loading control. *P < 0.05

UPR halts massive mRNA translation to relieve ER protein overload^19^, which may impair the expression of short-lived proteins. The short-lived protein FLIP inhibits caspase-8 processing and activation and causes TRAIL resistance. FLIP isoform FLIP_L_ played more important role than FLIP_S_ in preventing U87 cells from TRAIL-induced apoptosis (Supplementary Fig. S5A, B). KPNB1 knockdown or IPZ treatment downregulated both FLIP_L_ and FLIP_S_ in U87 cells. Overexpression of FLIP_L_ greatly while FLIP_S_ moderately reversed TRAIL-induced apoptosis in these cells (Fig. 5a, b), indicating KPNB1 inhibition downregulates FLIP to increase TRAIL sensitivity. KPNB1 inhibition decreased neither mRNA expression nor protein stability of FLIP (Supplementary Fig. S5C, D). Therefore, factors other than transcription, mRNA processing and protein turnover regulated FLIP expression. During the treatment of actinomycin D (Act D) and MG132 that inhibit transcription and protein degradation respectively, protein levels of FLIP_L_ and FLIP_S_ in control cells increased more than in KPNB1-inhibited U87 cells (Supplementary Fig. S5E), suggesting the change of FLIP protein synthesis. To verify the translational regulation of FLIP, we investigated the cap-dependent translation initiation signaling, in which 4E-BP1 and eIF2α are controlled by UPR to suppress cap-dependent translation initiation^19–22^. In addition to the upregulated p-eIF2α, KPNB1 knockdown or IPZ treatment in U87 cells upregulated α isoform of 4E-BP1 (4E-BP1α) that competed with eIF4G for binding m^7^GTP (mimicking mRNA 5′ cap)-bound eIF4E (Fig. 5c), thus impairing the integrity of cap-dependent translation initiation complex eIF4F. Therefore, IPZ treatment decreased total protein synthesis in U87 and U251 (Fig. 5d). Nevertheless, mTOR-regulated p-4E-BP1 (T37/46) which influences 4E-BP1 activity was not altered (Fig. 5c). 4E-BP1 knockdown reconstituted cap-bound eIF4E/eIF4G complex (Fig. 5e, f), restored FLIP_S_ levels (Fig. 5g, h) and nullified TRAIL-induced apoptosis (Fig. 5i, j) upon KPNB1 inhibition in U87 cells. Unexpectedly, 4E-BP1 knockdown suppressed FLIP_L_ expression (Fig. 5g, h). Considering 4E-BP1 does not regulate FLIP_L_ translation in glioblastoma cells^23^, we no longer investigated their relationship in the following study. ATF4-mediated KPNB1 inhibition-induced upregulation of 4E-BP1 transcript and 4E-BP1α protein (Fig. 5k, l), recapitulating the previous finding that ATF4 induced 4E-BP1 under ER stress^21^. However, ATF4 knockdown failed to rescue shKPNB1/TRAIL-induced apoptosis (Supplementary Fig. S6). EIF2α (S52A) overexpression suppressed IPZ-induced upregulation of ATF4 but not 4E-BP1α (Fig. 5g), possibly due to other unknown signalings regulated by the functional non-phosphorylated eIF2α. Moreover, bioinformatics analysis indicated that KPNB1 mRNA expression had moderate negative correlation with that of ATF4 in glioblastoma multiforme but not low grade glioma (Supplementary Fig. S7). These results suggest that KPNB1 inhibition upregulates 4E-BP1 by ATF4, which reduces the cap-dependent translation of FLIP_S_ and TRAIL resistance.

### KPNB1 inhibition-induced autophagy targets cleaved caspase-8 for clearance and counteracts apoptosis

**Fig. 6:**
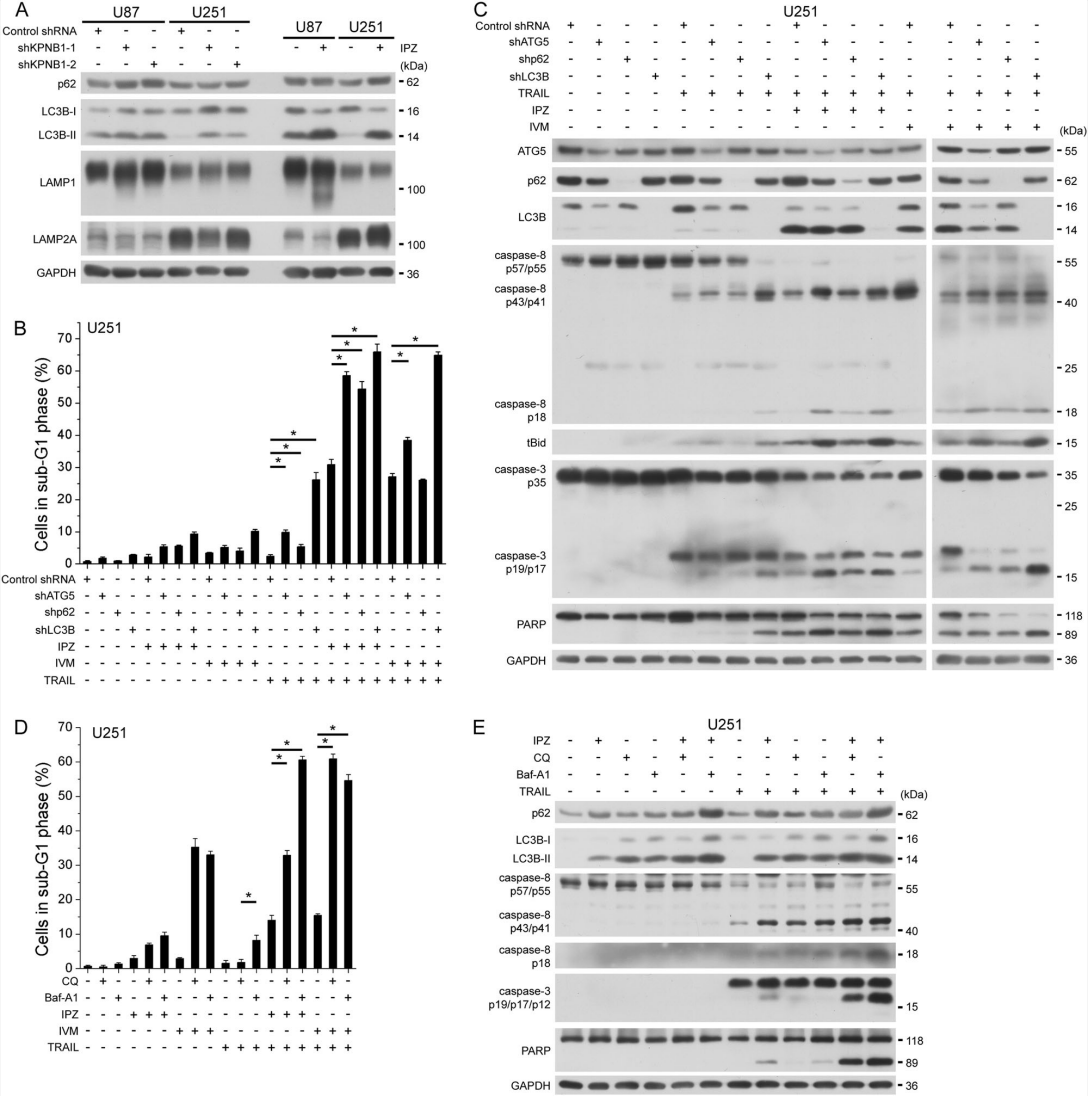
KPNB1 inhibition-induced autophagy restrains the cytotoxicity of TRAIL. **a** U87 and U251 cells expressing shKPNB1s or treated with IPZ (16 μM) for 24 h were subjected to western blot. **b**, **c** U251 cells expressing shATG5, shp62 or shLC3B were treated with IPZ (16 μM) or IVM (16 μM) for 24 h and further with TRAIL (100 ng/ml) for 6 h (western blot) or 24 h (flow cytometry), then subjected to flow cytometry (**b**) and western blot **c**. Results represent mean ± SD from three independent experiments. **d**, **e** U251 cells were pretreated with CQ (40 μM) or Baf-A1 (5 nM) along with IPZ (16 μM) or IVM (16 μM) for 24 h and further with TRAIL as in **b**, then subjected to flow cytometry (**d**) and western blot (**e**). Results represent mean ± SD from three independent experiments. GAPDH was used as the loading control. *P < 0.05

**Fig. 7:**
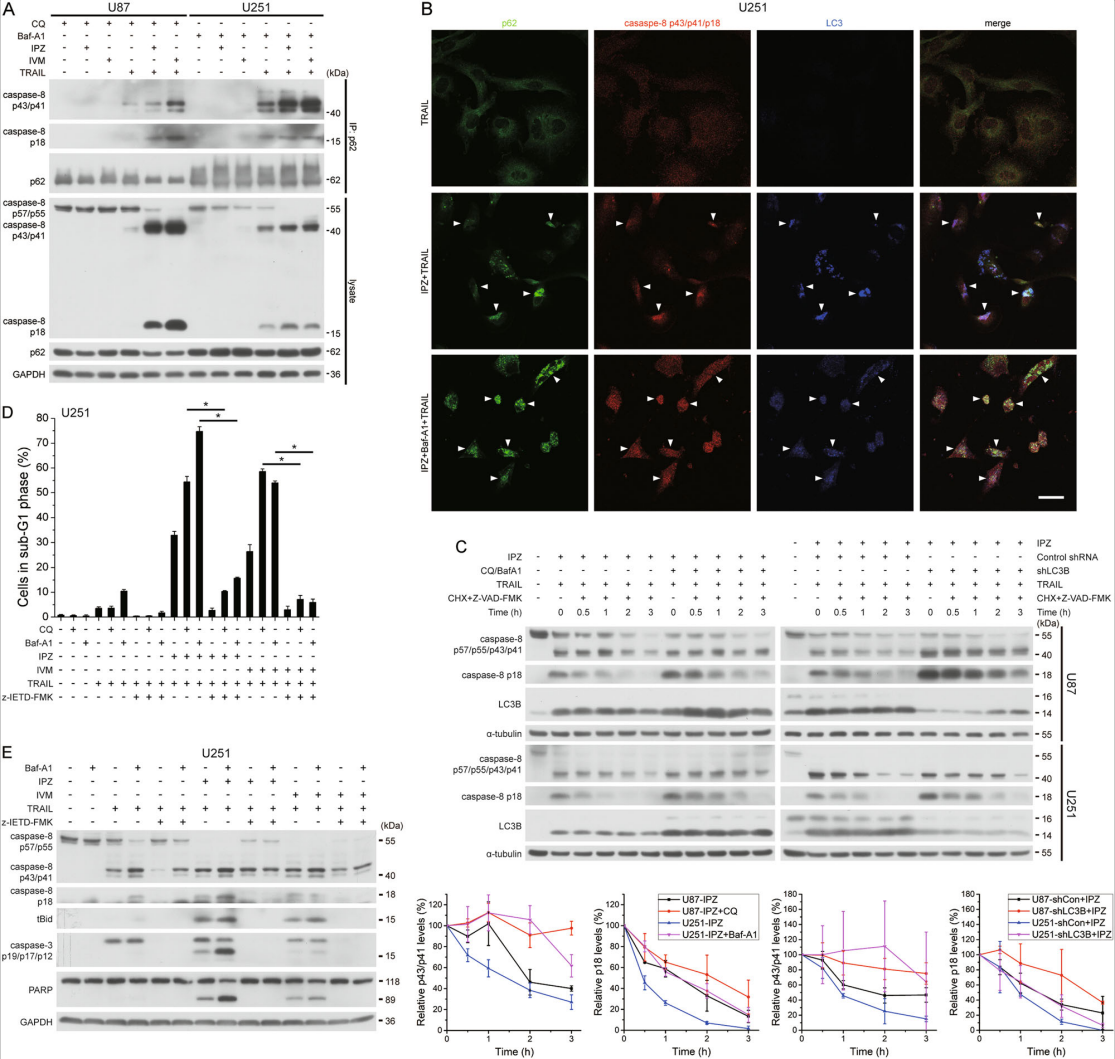
KPNB1 inhibition-induced autophagy eliminates TRAIL-induced cleaved caspase-8. **a** U87 and U251 cells were pretreated with CQ (40 μM) or Baf-A1 (5 nM) along with IPZ (16 μM) or IVM (16 μM) for 24 h and further with TRAIL (U87, 50 ng/ml; U251, 100 ng/ml) for 3 h. P62 in cell lysates was immunoprecipitated followed by western blot. **b** Representative images showed colocalization of p62, caspase-8 p43/p41/p18, and LC3 in U251 cells when treated with Baf-A1 (5 nM) and IPZ (16 μM) for 24 h and further with TRAIL (100 ng/ml) and z-DEVD-FMK (50 μM) for 8 h. Magnification, × 60; scale bar 25 μm. **c** U87 and U251 cells expressing shLC3B or not were treated with IPZ (16 μM) and CQ (40 μM) or Baf-A1 (5 nM) for 24 h and further with TRAIL (U87, 50 ng/ml; U251, 100 ng/ml) for 2 h. Then, cells were subjected to CHX pulse-chase assay by treating with CHX (U87, 20 μg/ml; U251 100 μg/ml) and z-VAD-FMK (20 μM) for indicated period of time. Representative images of western blot were shown in the upper panel. Quantification of grayscale ratio of cleaved caspase-8/α-tubulin by Photoshop software were shown in the lower panel. Results represent mean ± SD from two independent experiments. **d**, **e** U251 cells were pretreated as in **a** and further with z-IETD-FMK (20 μM) and TRAIL (100 ng/ml) for 6 h (western blot) or 24 h (flow cytometry), then subjected to flow cytometry (**d**) and western blot **(e**). Results represent mean ± SD from three independent experiments. GAPDH and α-tubulin was used as loading controls. *P < 0.05

KPNB1 knockdown or IPZ treatment activated autophagy, as revealed by the diffuse bands of the lysosome marker LAMP1 and upregulation of autophagy markers LC3B-II and/or p62 (Fig. 6a). Bioinformatics analysis supported a negative correlation between mRNA expression of KPNB1 and LC3B in glioblastoma multiforme (Supplementary Fig. S7). LC3B-II level that inversely correlates with autophagosome clearance was higher in U87 cells than in U251 cells with or without KPNB1 inhibition (Fig. 6a). Given that U251 cells were less sensitive to KPNB1 inhibition/TRAIL combination than U87 cells (Fig. 1b–e), we predicted that autophagic flux level somehow negatively correlated with the efficacy of such combination. We used IPZ and another KPNB1 inhibitor ivermectin (IVM), which has similar effects on UPR, protein synthesis and downstream molecules linked to TRAIL sensitivity as IPZ (Supplementary Fig. S8A and Fig. 5d) in the remainder study. Knocking down key autophagy regulators ATG5, p62, and LC3B all further enhanced apoptosis in U251 and U87 cells following KPNB1 inhibitor/TRAIL treatment (Fig. 6b, c and Supplementary Fig. S8B, C). Lysosome inhibitors chloroquine (CQ) or bafilomycin A1 (Baf-A1) had similar effect on apoptosis induction (Fig. 6d, e and Supplementary Fig. S8D-F). These results substantiate a pro-survival role of autophagy in KPNB1 inhibitor/TRAIL treatment. In U87 and U251 cells, both knockdown of ATG5, p62 or LC3B and treatment of CQ or Baf-A1 upregulated the intermediate product p43/p41 and the active product p18 of cleaved caspase-8, which facilitated the cleavage of Bid, caspase-3 and PARP (Fig. 6c, e and Supplementary Fig. S8C, E). Among them, p62 knockdown had the least effect on cleaved caspase-8 accumulation and apoptosis promotion (Fig. 6b, c and Supplementary Fig. S8B, C), possibly because procaspase-8 requires p62 for aggregation and cleavage upon TRAIL stimulation^24^. After cleavage caspase-8 p43/p41 and p18 products are ubiquitinated for degradation and colocalized with the autophagolysosome probably for clearance^25^. KPNB1 inhibitor/TRAIL combination-induced p62 binding to p43/p41 and p18 in U87 and U251 cells and colocalization of p62, p43/p41/p18 and LC3 in U251 cells (Fig. 7a, b). Consistently, blocking lysosomal degradation with lysosome inhibitor or LC3B knockdown stabilized caspase-8 p43/p41 and p18 (Fig. 7c). Caspase-8 inhibitor Z-IETD-FMK reversed cell apoptosis induced by KPNB1 inhibitor, TRAIL and autophagy inhibition (Fig. 7d, e and Supplementary Fig. S8H-J). Thus, blocking autophagy-lysosomal degradation prevents cleaved caspase-8 from clearance and markedly augmented the synergism of KPNB1 inhibitor/TRAIL combination in glioblastoma cells.

### Combination of IVM, CQ, and TRAIL reduces tumor growth in vivo

**Fig. 8:**
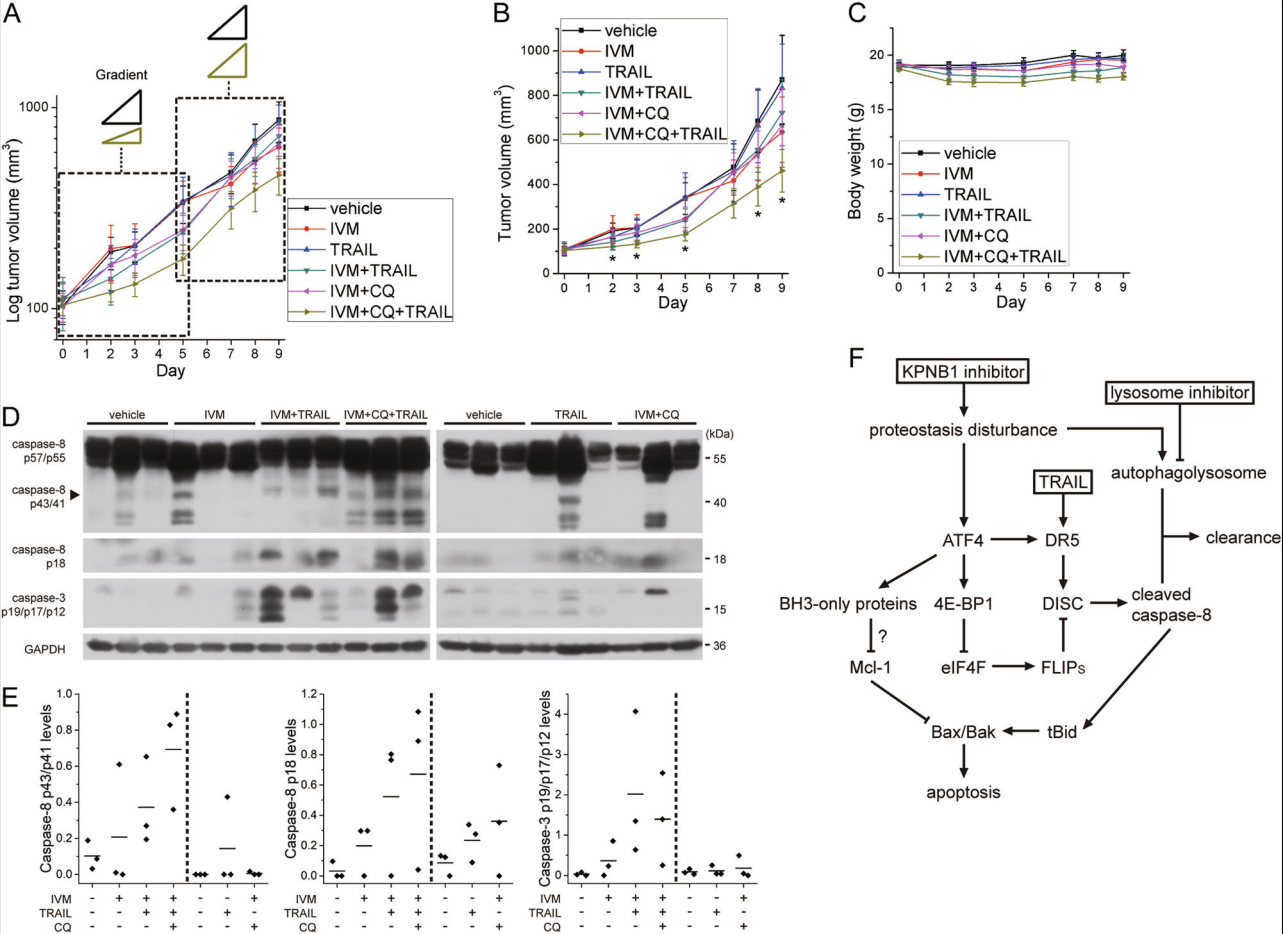
Combination of IVM, CQ and TRAIL suppresses tumor growth in vivo. **a** Log-transformed tumor volume curves of U87 xenografts in mice treated with IVM (1.5 mg/kg, intraperitoneal injection, daily, from day 0 to 8), CQ (50 mg/kg, intraperitoneal injection, daily, from day 0 to 8), TRAIL (100 μg, intraperitoneal injection, daily, from day 1 to 8), their solvents or indicated combination (*n* = 7 except IVM + CQ + TRAIL group is *n* = 8, mean ± SEM). **b, c** Tumor volume curves (**b**) and body weight curves (**c**) of treated U87 xenografts (mean ± SEM). *P < 0.05 compared with vehicle group. **d** Western blot analysis of U87 xenografts isolated from mice treated as indicated. GAPDH was used as the loading control. **e** Quantification of grayscale ratio of cleaved caspase-8 or cleaved caspase-3 to GAPDH in **d** by Photoshop software. The line represent the mean value. **f** Schematic overview of the purposed pathway for the combination of KPNB1 inhibitor, TRAIL and lysosome inhibitor based on the results of this study

At last, we analyzed the anticancer activity of combination of KPNB1 inhibitor, lysosome inhibitor, and TRAIL in nude mice subcutaneously inoculated U87 cells. We purified sufficient amount of human recombinant soluble TRAIL, which is trimeric in PBS solution (Supplementary Fig. S9A, B). IVM with proved clinical safety was used as KPNB1 inhibitor for the therapy. IVM, CQ plus TRAIL better inhibited tumor growth than vehicle, single-treatment or double-treatment in early stage (day 1–5) but not in later stage (day 5–9) according to gradients of log-transformed tumor volume curves (Fig. 8a). The mean tumor volume of IVM + CQ + TRAIL group is 53% of that of vehicle group (Fig. 8b). Combination of IVM, CQ, and TRAIL was tolerated, with moderate weight loss late in the treatment (Fig. 8c). IVM and/or CQ enhanced TRAIL-induced cleavage of caspase-8 and caspase-3 in tumors (Fig. 8d, e). These results suggest that combination of IVM, CQ, and TRAIL reduces xenograft growth by inducing apoptosis. Nevertheless, TRAIL dosage (100 μg) in this therapy was too low to synergize with IVM and/or CQ to reduce tumor growth and activate death receptor signaling (in terms of procaspase-8/cleaved caspase-8 ratio) as potent as those in vitro, especially when tumors were big. Raising TRAIL dose or replacing it with modified TRAIL to improve its activity or pharmacokinetic profile^26,27^ may solve this problem.

## Discussion

In the present study, we delineate the molecular basis of KPNB1 inhibition in modulating TRAIL vulnerability of glioblastoma cells. Consistent with previous study, chemical or genetic inhibition of KPNB1 primes cells to TRAIL-induced apoptosis. However, rather than simply upregulating the cell surface DR5, KPNB1 inhibition triggers UPR to rewire the TRAIL receptor signaling and abrogate TRAIL resistance (Fig. 8f).

Earlier we found that KPNB1 inhibition in glioblastoma cells perturbed proteostasis and consequently activated UPR to alleviate stressful condition and to induce apoptosis of cells under chronic stress^9^. Therapies that activate UPR can sensitize cancer cells but not non-neoplastic cells to TRAIL treatment mainly through the following mechanisms. First, it upregulates DR5 expression commonly through the transcriptional factor CHOP and sometimes through ATF4 or IRE1α-XBP1s axis^28–30^. Sometimes increased DR4 expression dominates apoptosis by TRAIL and UPR^29,31^. Second, it upregulates pro-apoptotic Bcl-2 proteins like Bim and Puma, which block the anti-apoptotic Bcl-2 proteins^32^ to facilitate MOMP. Third, it inhibits translation initiation to downregulate proteins like Mcl-1, which block the death receptor signaling^33–35^. Although the expression of TRAIL receptors does not correlate with TRAIL sensitivity of cancer cells in general^1,4^, ample evidence supports that upregulating TRAIL receptors overcomes TRAIL resistance^15,16,32^. Like many UPR sensitizers, KPNB1 inhibition enhances ATF4-dependent DR5 transcription. DR5 upregulation is less dependent on CHOP because KPNB1 inhibition causes cytosolic retention of UPR-upregulated CHOP^9^. KPNB1 inhibition stabilizes DR5 by mechanisms beside c-Cbl downregulation. As a result, DR5 upregulation increases cell surface DR5 level, DISC formation and apoptotic signaling transduction upon KPNB1 inhibition and TRAIL treatment. On the other hand, KPNB1 inhibition sometimes upregulates total DR4 but does not commonly affect cell surface DR4 level. DR4 is lowly expressed in glioblastoma cells and less important for apoptosis by TRAIL and KPNB1 inhibition. KPNB1 inhibition promotes Noxa expression and Noxa/Mcl-1 interaction, which may free Bak and Bax from Mcl-1 to induce MOMP. However, Noxa upregulation is dispensable for IPZ/TRAIL-induced apoptosis possibly owing to the redundancy and abundance of BH3-only proteins in neutralizing Mcl-1.

KPNB1 inhibition downregulates FLIPs by suppressing cap-dependent translation initiation. One aim of PERK branch activation is to alleviate global protein synthesis by p-eIF2α to relieve UPR^19^. Besides, changes in mTOR-4E-BP1 axis upon UPR also suppress translation^36^. KPNB1 inhibition does not decrease p-mTOR and p-4E-BP1 but increases ATF4-mediated 4E-BP1 expression to inhibit FLIP_S_ translation. KPNB1 inhibition-induced p-eIF2α upregulates ATF4 but not downstream 4E-BP1, thereby leaving FLIP_S_ expression unaffected. Moreover, ATF4 knockdown does not rescue KPNB1 inhibition/TRAIL-induced apoptosis possibly because ATF4 is required for Mcl-1 transcription under UPR^37^. The translation rates of FLIP_S_ and FLIP_R_ vary^38^, but those of FLIP_S_ and FLIP_L_ are unknown. Our study showed that within the time both mRNA production and protein degradation are inhibited, FLIP_S_ protein accumulates more than FLIP_L_ protein. Moreover, FLIP_L_ translation is irrelevant to 4E-BP1 activity but sometimes is controlled by mTOR-S6K1 axis^23,39^. Therefore, translation modes of FLIP_S_ and FLIP_L_ differ. We speculate that 4E-BP1 knockdown may unleash FLIP_S_ translation, which competes with FLIP_L_ translation for resources and further limits FLIP_L_ expression. We still need to know how FLIP_L_ is downregulated as it is necessary for KPNB1 inhibition/TRAIL-induced apoptosis.

KPNB1 inhibition-induced autophagy protects glioblastoma cells from proteotoxicity^9^. The role of autophagy in TRAIL receptor signaling is more complicated. Autophagy facilitates incipient TRAIL receptor complex trafficking and caspase-8 activation^40,41^. However, evidences also support that autophagy negatively regulates TRAIL-triggered apoptosis. Cells with high autophagic flux and low p62 aggregation are resistant to TRAIL^42^. Autophagosome also impairs membrane localization of death receptors causing TRAIL resistance^43^. Upon TRAIL stimulation, the ubiquitin-proteasome system degrades cleaved caspase-8^44^. However, whether autophagy degrades cleaved caspase-8 to avoid TRAIL-induced apoptosis is uncertain, though capsase-8 aggregates colocalize with autophagosomes and lysosomes upon TRAIL stimulation^25^. In this study, genetic inhibition of autophagosome biogenesis or extension or pharmacological inhibition of lysosomal degradation further augments cleaved caspase-8 level and consequent apoptosis upon KPNB1 inhibitor/TRAIL treatment, which is abrogated by caspase-8 inhibitor. Blocking lysosomal degradation stabilizes cleaved caspase-8 with minor effect on procaspase-8 cleavage. Mechanistically, p62 binds caspase-8 p43/p41 and p18 following lysosome inhibitor and TRAIL treatment and binds more with KPNB1 inhibitor cotreatment. In concert with previous findings^25^, aggregated cleaved caspase-8, p62, and LC3 colocalized after KPNB1 inhibitor/TRAIL treatment. Thus, p62-labeled cleaved caspase-8 is targeted by autophagosome for degradation, which disturbs KPNB1 inhibitor/TRAIL-triggered apoptosis. These data challenge the hypothesis that accumulation of p62 aggregates upon autophagy inhibition causes cytotoxicity or facilitates aggregation and cleavage of procaspase-8 to upregulate cleaved caspase-8^42^, as p62 knockdown promoted three drug combination-induced apoptosis. These results underline that autophagy favors initial caspase-8 activation under TRAIL stimulation or cytotoxic stresses^45^ but impacts more on caspase-8 turnover lowering TRAIL response.

Overall, our findings provide a potentially novel anticancer mechanism of combining TRAIL, KPNB1 inhibitor and lysosome inhibitor. Combination of KPNB1 inhibitor and TRAIL may be applicable for treating tumors other than glioblastoma in view of data from previous study^8^ and ours (Supplementary Fig. S10A, B) but demands further exploration. Given that TRAIL, IVM and CQ show clinical safety, such combination therapy may rapidly move into anticancer clinical trials.

## Materials and methods

### Cell culture

Human glioblastoma cell lines U87, U118, U251, and A172 were cultured in DMEM supplemented with 10% fetal bovine serum, 1% non-essential amino acid, and 1% sodium pyruvate (Life technologies, Grand Island, USA). Human fetal astrocytes from cerebral cortex were cultured in astrocyte medium (ScienCell Research Labortories, Carlsbad, USA) with 2% fetal bovine serum. U87 and U251 were purchased in April 7, 2017 (purchase order, 85676) and U118 and A172 were purchased in July 13, 2018 (purchase order, 115354) from cell bank of Chinese Academy of Sciences (Shanghai, China), where they were authenticated by means of STR profiling. Human astrocytes were obtained from ScienCell Research Laboratories. All cells were maintained under standard cell culture conditions at 37 °C and 5% CO_2_.

### Antibodies and reagents

Primary antibodies used in this study were listed below: antibodies against α-tubulin (HRP-conjugated) (HRP-66031), KPNB1 (10077–1-AP) (ProteinTech Group, Wuhan, China), LAMP2A (ab125068) (Abcam, Canbridge, UK), 4E-BP1 (9644), ATF4 (11815), ATG5 (12994), Bax (5023), Bak (12105), Bid (2002), caspase-3 (9662), caspase-8 (9746) (for western blot and immunoprecipitation), CHOP (2895), cleaved caspase-8 (9496), DR4 (42533), DR5 (8074), eIF4E (2067), eIF4G (2469), FLIP (56343), LAMP1 (9091), LC3A/B (Alexa Fluor 647) (13394), LC3B (3868), Mcl-1 (5453), MeCP2 (3456), mouse mAb IgG1 isotype control (5415) (for functional neutralization), Noxa (14766), p-4E-BP1 (T37/46) (2855), p62 (8025), p62 (Alexa Fluor 488) (8833), PARP (9532), Puma (12450) (Cell Signaling Technology, Beverly, USA), caspase-8 (sc-6136) (for detection of caspase-8 in caspase-8 immunoprecipitant) (Santa Cruz Biotechnology, Santa Cruz, USA), c-Cbl (610441), FADD (556402), Mcl-1 (for immunoprecipitation) (559027) (BD Biosciences, San Jose, USA), ATF3 (BS2261), DR4 (BS2238), eIF2α (BS3651), p-eIF2α (S51) (BS4787) (Bioworld Technology, Nanjing, China), Lamin B (BA1228) (Boster, Wuhan, China), GAPDH (KC-5G5) (Kangchen, Shanghai, China), APC anti-human CD261 (DR4, TRAIL-R1) (307208), APC anti-human CD262 (DR5, TRAIL-R2) (307408), APC mouse IgG1, κ isotype ctrl antibody (400119) (all for flow cytometry) (BioLegend, San Diego, USA), anti-TRAIL-R1 (human), mAb (HS101) (AG-20B-0022PF) and anti-TRAIL-R2 (human), mAb (HS201) (AG-20B-0023PF) (both for functional neutralization) (AdipoGen AG, Liestal, Switzerland). Anti-mouse (7076) or anti-rabbit (7074) secondary antibodies (horseradish peroxidase-conjugated) were acquired from Cell Signaling Technology. Alexa Fluor 546 goat anti-rabbit antibody (A-11010) was from Life Technologies (Carlsbad, USA).

Reagents and kits used in this study were listed below: importazole (IPZ) (Merck Millipore, Darmstadt, Germany), PMSF, propidium iodide (Sigma-Aldrich, St. Louis, USA), CalPhos Mammalian Transfection Kit (TaKaRa Bio, Kusatsu, Japan), protease inhibitor cocktail, RNase A, Click-iT™ Plus OPP Protein Synthesis Assay Kit Alexa Fluor™ 488 picolyl azide (Thermo Scientific, Waltham, USA), phosphatase inhibitor, thiazolyl blue tetrazolium bromide (Sangon, Shanghai, China), cell lysis buffer for Western and IP, cycloheximide (CHX), nuclear and cytoplasmic protein extraction kit, RIPA (Beyotime, Nantong, China), LightCycler 480 SYBR Green I Master, protein A agarose, protein G agarose (Roche Diagnositics, Indianapolis, USA), bafilomycin A1 (Baf-A1), ivermectin (IVM), MG132, z-VAD-FMK (Selleck, Shanghai, China), Act D, chloroquine diphosphate (CQ), z-DEVD-FMK, z-IETD-FMK (Medchem Express, Monmouth Junction, USA), human recombinant TRAIL (PeproTech, Rocky Hill, USA), iScript Reverse Transcription Supermix (Bio-Rad, Berkeley, USA), immobilized 2′/3′-EDA-m^7^GTP (Jena Bioscience, Jena,Germany) were used in this study.

### Lentivirus-mediated gene transduction

Short hairpin RNAs (shRNAs) targeting human KPNB1, DR5, ATF4, CHOP, Noxa, 4E-BP1, ATG5, p62, LC3B, and a scrambled (control) shRNA were inserted into the lentiviral vector pLKD-CMV-GFP-U6-shRNA. Coding DNAs of human c-Cbl, non-degradable Mcl-1 (T92A) mutant, FLIP_L_, FLIP_S_ and eIF2α (S52A) were inserted into pCDH-CMV-MCS-EF1-copGFP. Lentiviral plasmids, gag/pol packaging vector and VSVG encoding plasmid were transfected into 293T cells using CalPhos Mammalian Transfection Kit according to the manufacturer’s protocol. Culture medium was harvested 48 h and 72 h after transfection and ultracentrifugated to obtain high-titer purified preparation. The sequences of shRNAs used were listed in the Supplementary Data.

### Western blot

After collection, cells were lysed in RIPA supplemented with PMSF, phosphatase inhibitor, and protease inhibitor cocktail. Western blot was carried out as previously described^46^. Grayscale of protein bands was analyzed by Photoshop CS4 software.

### MTT assay

Cell viability was measured by MTT assay performed as previously described^46^.

### Flow cytometry assay

To measure cell surface DR4 and DR5 expression, intact cells were stained with APC anti-human DR4 or DR5 antibody or IgG isotype ctrl antibody for 30 min at 4 °C. To measure apoptosis, fixed cells were stained with propidium iodide as previously described^46^. After staining, cells were analyzed using a BD LSR II flow cytometer. Sub-diploid cells were considered apoptotic. The mean fluorescence intensity and proportion of sub-dipliod cells were analyzed by FlowJo 7.6.1 software.

### Immunoprecipitation and DISC analysis

Cells were lysed on ice in cell lysis buffer for Western and IP (20 mM Tris (pH 7.5), 150 mM NaCl, 1% Triton X-100). Lysates were adjusted to have equal protein concentrations and incubated with indicated antibodies overnight, and with additional protein A agarose or protein G agarose at 4 °C for 3 h. For DISC analysis, cells were treated with 20 μM z-VAD-FMK for 5 h and further with or without 100 ng/ml TRAIL for 1 h. Cells were lysed in lysis buffer (50 mM Tris-HCl (pH 7.5), 150 mM NaCl, 1% Triton X-100, 1 mM EDTA, 10% glycerol). Lysates were incubated with caspase-8 (9746) antibody overnight to immunoprecipitate DISC. Precipitates were washed three times with lysis buffer before adding SDS-PAGE loading buffer and denaturation. Precipitates and lysates were then subjected to western blot.

### RNA extraction and real-time PCR

RNA was extracted, reverse transcribed and analyzed by quantitative real-time PCR. Relative gene mRNA levels were normalized to that of GAPDH. The comparative Ct method was used to calculate fold changes in expression. Primer sets for PCR were obtained from PrimerBank are listed in the Supplementary Data.

### Cap-binding assay

Cell lysates were incubated with m^7^GTP agarose at 4 °C for 3 h to capture eIF4E and its binding proteins. Following procedures were performed in the same procedure as immunoprecipitation.

### Protein synthesis assay

Cells were plated in 96-well culture plates. After treatment with indicated drugs, cells were incubated with 20 μM O-propargyl-puromycin (OPP) for 0.5 h. OPP detection and normalization to cell number were performed according to the manufacturer’s protocol. FITC intensity was measured by multi-mode microplate reader.

### Immunofluorescence

Cells cultured onto coverslips were fixed with 4% paraformaldehyde at room temperature for 15 min. Cells were incubated with blocking buffer (5% bovine serum albumin and 0.1% Triton in PBS) for 1 h and stained with antibodies and Hoechst 33342 at 4 °C overnight. Fluorescent signals were observed with a Nikon FN1 confocal microscope at × 60 magnification.

### Expression and purification of recombinant TRAIL

The cDNA sequence encoding the extracellular portion of human TRAIL (aa 114–281) was inserted into pGEX-6p-1. Recombinant GST-TRAIL was expressed in Rosetta bacteria as the expression host. The overnight seed culture was diluted 500-fold into LB broth and incubated at 37 °C. When OD_600_ of the broth is around 0.6–1, isopropyl-1-thio-β-D-galactopyranoside (0.5 mM) was added to induce recombinant protein expression, and the culture was incubated at overnight 25 °C. Bacteria were harvested and resuspended in PBS + 0.5% Tween 20 + proteasome inhibitor and then cracked by high pressure homogenizer. Recombinant GST-TRAIL was isolated from the soluble fraction by affinity chromatography using Glutathione-Sepharose 4B and soluble TRAIL was seperated by prescission protease. The purity of recombinant TRAIL was confirmed by SDS-PAGE and Coomassie Blue staining. The oligomeric state was analyzed by gel filtration chromatography. The protein concentration was calculated by comparing the grayscale of its band with that of BSA standard.

### In vivo study

Female BALB/c nude mice (5 weeks, Shanghai Lingchang Bioscience Company, China) were maintained in the pathogen-free environment. All experimental procedures were approved by the Institutional Animal Care and Use Committee of the Institute of Neuroscience, Chinese Academy of Sciences. U87 cells (5 × 10^6^) in 150 μl serum-free DMEM were inoculated subcutaneously into the area under the left flank of mice. When average tumor volume reached approximately 100 mm^3^, mice were randomized into six groups (*n* = 7 except IVM + CQ + TRAIL group is *n* = 8): vehicle, IVM, TRAIL, IVM + TRAIL, IVM + CQ, IVM + CQ + TRAIL. Mice were pretreated with IVM (6 mg/kg, in 1.5% DMSO + 58.5% 1, 2-propylene glycol + 40% water), CQ (50 mg/kg, in saline) or solvents by intraperitoneal injection. One day later, mice were treated with IVM, CQ, TRAIL (100 μg, in PBS + 2% Tween 20) or solvents by intraperitoneal injection daily for 8 days. Tumor length and width were measured by caliper. Tumor volume was calculated using the formula: V = 0.5 × length × width^2^. One day after the final treatment, mice were euthanized and tumors were immediately isolated and lysed for western blot.

### Statistical analysis

OriginPro 8 software (OriginLab Corporation, Northampton, USA) was used for data analysis and graphing. Results are expressed as mean ± SD unless indicated. The two-tailed unpaired t-test was used to determine significant differences between the mean values of groups, with statistical significance defined as *p* < 0.05.

## Supplementary information

Supplementary Information
