# Supplementary material for: Targeting KPNB1 overcomes TRAIL resistance by regulating DR5, Mcl-1 and FLIP in glioblastoma cells
Source: Cell Death Dis. 2019 Feb 11;10(2):118. doi: 10.1038/s41419-019-1383-x (PMC6370806; doi:10.1038/s41419-019-1383-x)
Supplement: Supplementary file 1 — Supplementary Information [file 41419_2019_1383_MOESM1_ESM.pdf]

Supplementary Information

The sequence of shRNAs used were as follows: shKPNB1-1, CCGGGGAAGTGTTGGGTGGTGAATTCTCGAGAATTCACCACCCAACACTTCCTTTTTTG; shKPNB1-2, CCGGGGAAGTGTTGGGTGGTGAATTCTCGAGAATTCACCACCCAACACTTCCTTTTTTG; shDR5, CCGGCTGATAAAGTGGGTCAACATTCAAGAGATGTTGACCCACTTTATCAGTTTTTTG; shATF4, CCGGGGATAGTCAGGAGCGTCAATGCTCGAGCATTGACGCTCCTGACTATCCTTTTTTG; shCHOP, CCGGGCCAATGATGTGACCCTCAATCTCGAGATTGAGGGTCACATCATTGGCTTTTTTG; shNoxa, CCGGGCAAGAACGCTCAACCGAGTTCAAGAGACTCGGTTGAGCGTTCTTGCTTTTTTG; shFLIP$_L$, CCGGGAGCTTCTTCGAGACACCTTCCTCGAGGAAGGTGTCTCGAAGAAGCTCTTTTTTG; shFLIP$_S$, CCGGGCCAAGCAGTTCTTAACATTTCTCGAGAAATGTTAAGAACTGCTTGGCTTTTTTG; sh4E-BP1, CCGGGCCAGGCCTTATGAAAGTGATCTCGAGATCACTTTCATAAGGCCTGGCTTTTTT; shATG5, CCGGTCATGGAATTGAGCCAATGTTCTCGAGAACATTGGCTCAATTCCATGATTTTTTG; shp62, CCGGGGAACAGATGGAGTCGGATAACTCGAGTTATCCGACTCCATCTGTTCCTTTTTTG; shLC3B, CCGGGCTTACAGCTCAATGCTAATCCTCGAGGATTAGCATTGAGCTGTAAGCTTTTTTG; scrambled shRNA, CCGGTTCTCCGAACGTGTCACGTTTCAAGAGAACGTGACACGTTCGGAGAATTTTTTG.

Primer sets used for real-time PCR were as follows: human ATF4, 5’-CCCTTCACCTTCTTACAACCTC-3’, 5’-TGCCCAGCTCTAAACTAAAGGA-3’; human DR5, 5’-GCCCCACAACAAAAGAGGTC-3’, 5’-AGGTCATTCCAGTGAGTGCTA-3’; human CHOP, 5’- GGAAACAGAGTGGTCATTCCC-3’, 5’-CTGCTTGAGCCGTTCATTCTC-3’; human FLIP, 5’- AGAGTGAGGCGATTTGACCTG-3’, 5’-GTCCGAAACAAGGTGAGGGTT-3’; human 4E-BP1, 5’-CTATGACCGGAAATTCCTGATGG-3’, 5’-CCCGCTTATCTTCTGGGCTA-3’; human GAPDH, 5’-CCCCAATGTATCCGTTGTG-3’, 5’-CTCAGTGTAGCCCAGGATGC-3’.

Supplementary Figures

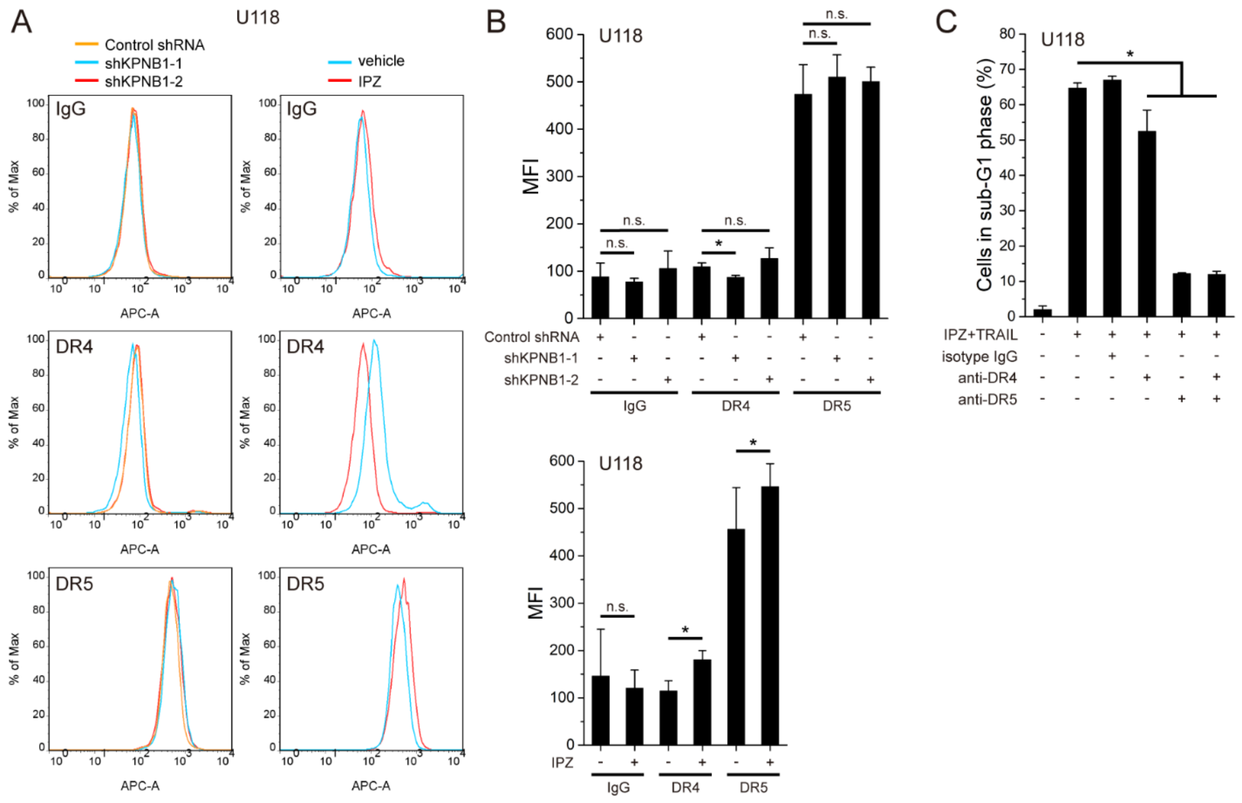

Supplementary Figure S1. (**A, B**) Intact U118 cells either expressing shKPNB1s or treated with IPZ (16 μM) for 24h were stained with APC anti-human DR4 or DR5 antibody or IgG isotype ctrl antibody. Cell surface DR4 or DR5 levels were measured by flow cytometry **A**. MFI was shown in **B**. Results represent mean±SD from three (shKPNB1s) or five (IPZ) independent experiments. **P* < 0.05. n.s., not significant. (**C**) U118 cells pretreated with IPZ (16 μM) for 24 h were treated with antagonistic antibody to DR4 or DR5 or IgG1 isotype control antibody (all 5 μl/ml) for 1 h and further with TRAIL for 24 h, then subjected to flow cytometry. Results represent mean±SD from three independent experiments. **P* < 0.05.

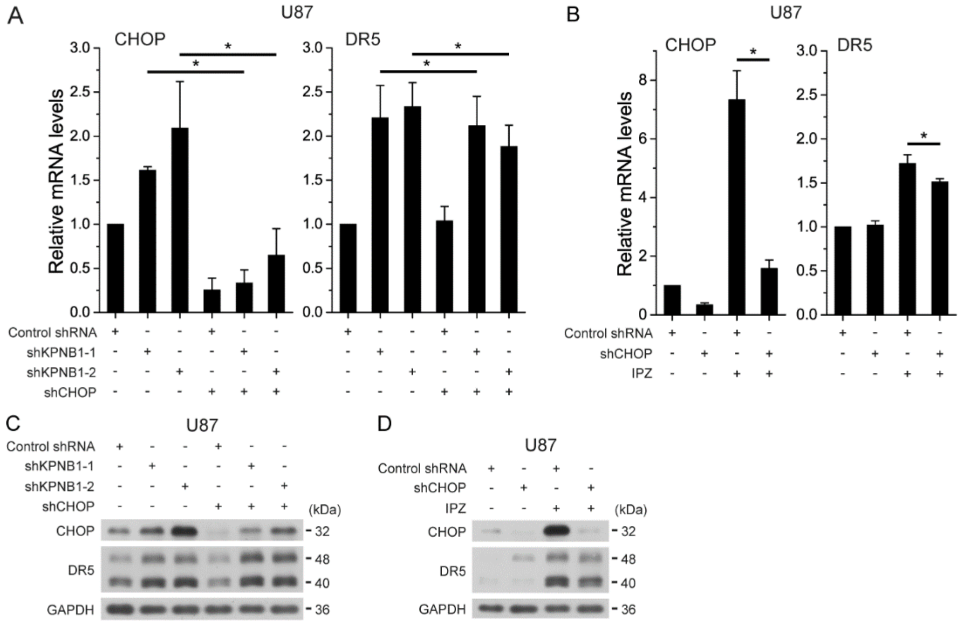

Supplementary Figure S2. (**A**, **B**) Real-time PCR analysis of indicated genes in U87 cells expressing shKPNB1s and/or shCHOP **A**, and in U87 cells expressing shCHOP and/or treated with IPZ (16 μM) **B**. Results represent mean±SD from three independent experiments. *$P < 0.05$. (**C**, **D**) Western blot analysis of indicated proteins in U87 cells treated as in **A** and **B**. GAPDH was used as the loading control.

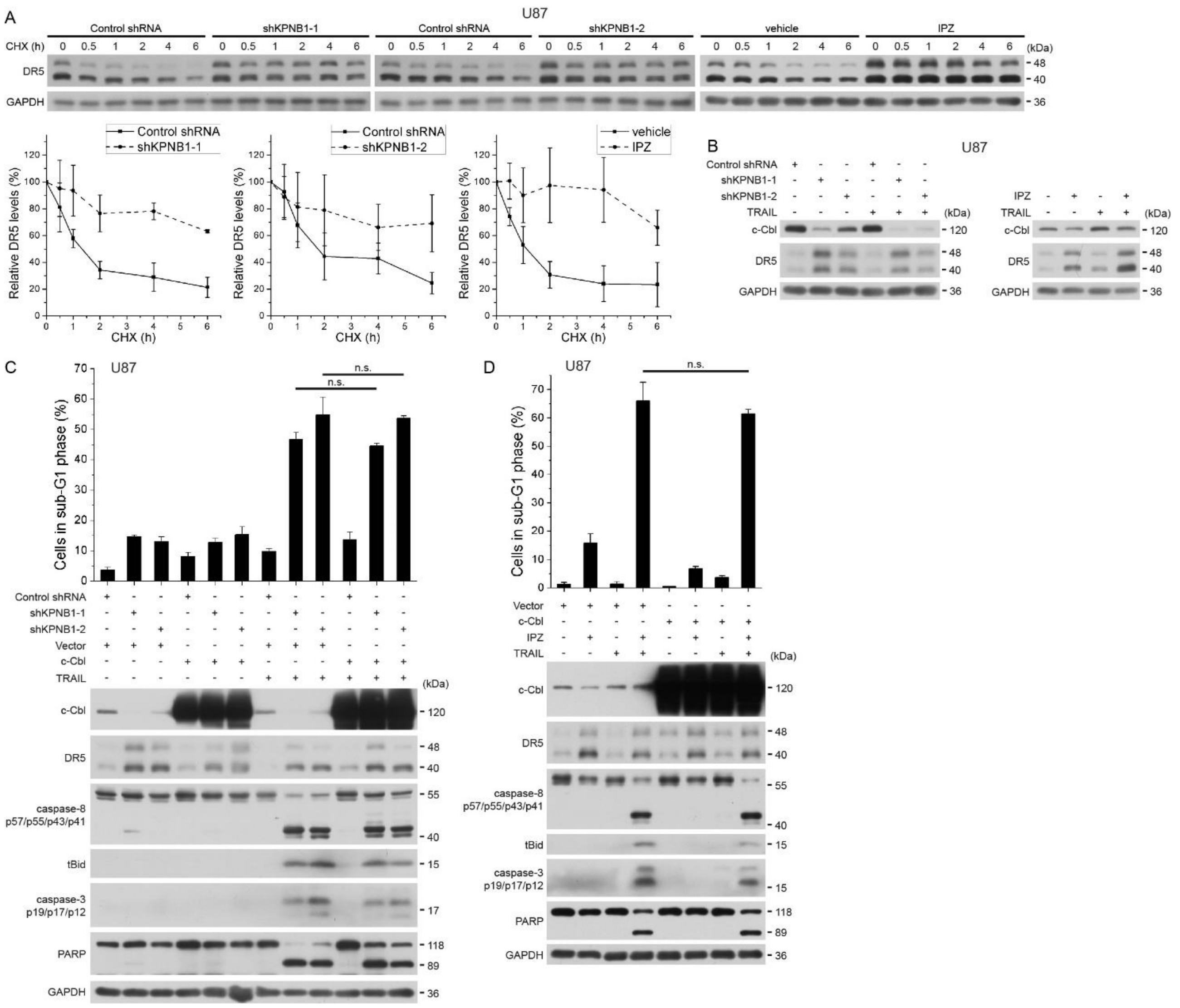

Supplementary Figure S3. (**A**) shKPNB1-expressing or IPZ (16 μM)-treated U87 cells were subjected to cycloheximide (CHX) pulse-chase assay. Representative images of western blot were shown in the upper panel. Quantification of grayscale ratio of DR5/GAPDH by Photoshop software were shown in the lower panel. Results represent mean±SD from three (shKPNB1) or two (IPZ) independent experiments. (**B**) Western blot analysis of indicated proteins in U87 cells expressing shKPNB1s or pretreated with IPZ (16 μM) for 24h and further treated with TRAIL. (**C**) U87 cells expressing shKPNB1s and/or c-Cbl were treated with TRAIL and subjected to western blot or flow cytometry. Results represent mean±SD from three independent experiments. (**D**) U87 cells expressing c-Cbl were treated with IPZ (16 μM) for 24h and further with TRAIL, then subjected to western blot and flow cytometry. Results represent mean±SD from three independent experiments. GAPDH was used as the loading control. **P*< 0.05; n.s., not significant.

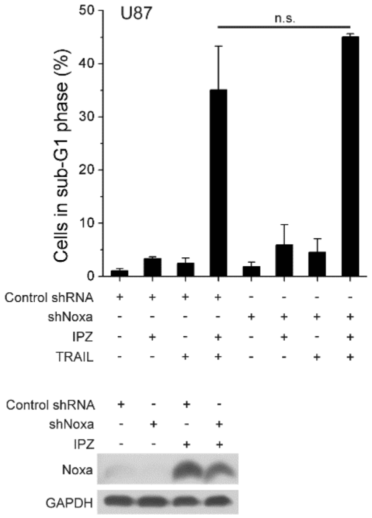

Supplementary Figure S4. U87 cells expressing shNoxa were treated with IPZ (16 μM) for 24h and further with TRAIL (30 ng/ml) for 24h, then subjected to flow cytometry. Results represent mean±SD from three independent experiments. n.s.: not significant. Knockdown efficacy of shNoxa in U87 cells with or without IPZ treatment was analyzed by western blot. GAPDH was used as the loading control.

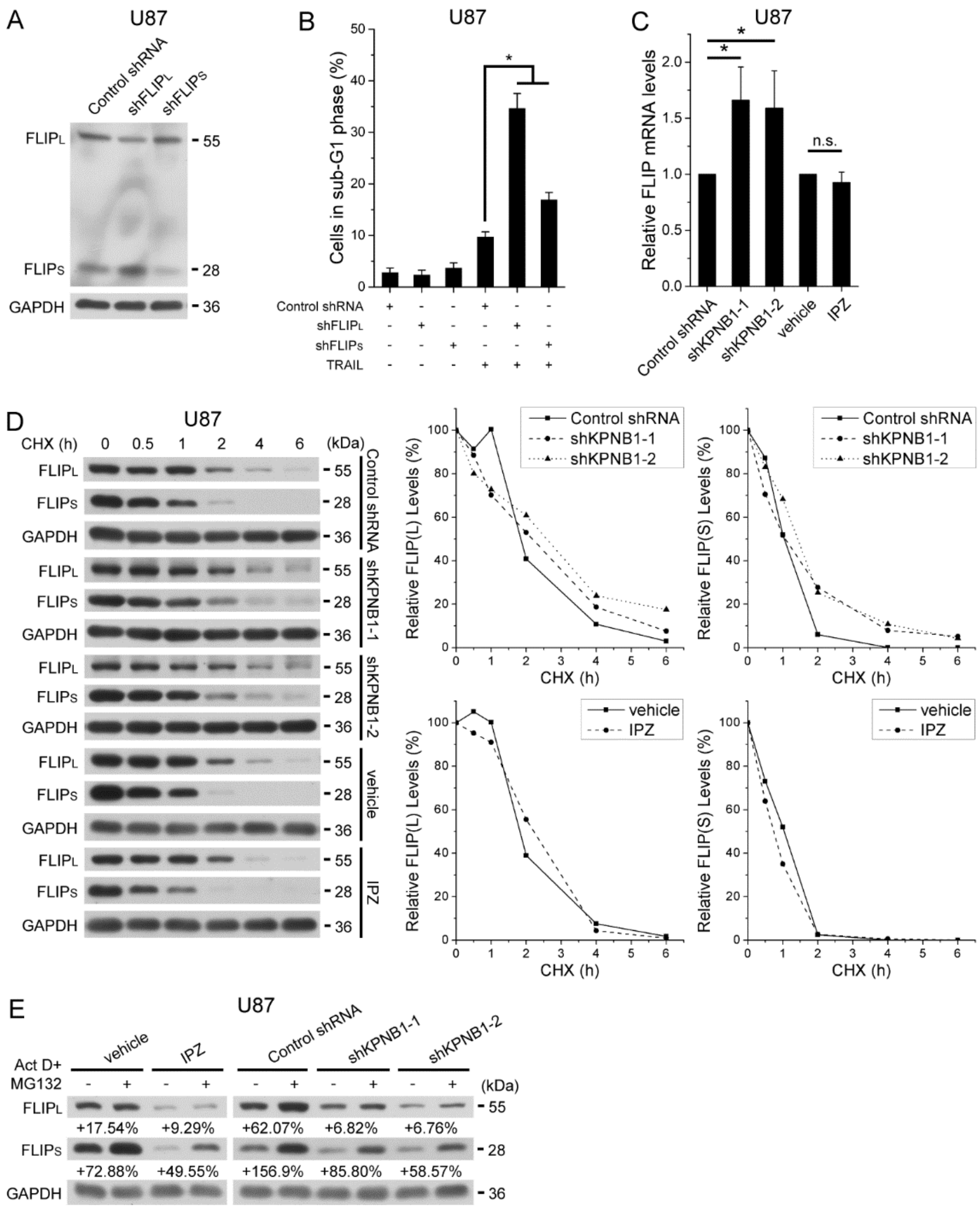

Supplementary Figure S5. (**A**) Knockdown efficacy of shFLIP$_L$ and shFLIP$_S$ in U87 cells was validated by western blot. (**B**) U87 cells expressing shFLIP$_L$ or shFLIP$_S$ were treated with TRAIL (30 ng/ml) for 24 h and subjected to flow cytometry. Results represent mean±SD from three independent experiments. **P*< 0.05. (**C**) Real-time PCR analysis of FLIP mRNA expression in U87 cells expressing shKPNB1s or treated with IPZ (16 μM) for 24h. Results represent mean±SD from three (shKPNB1s) or two (IPZ) independent experiments. **P*< 0.05; n.s.: not significant. (**D**) U87 cells expressing shKPNB1s or pretreated with IPZ (16 μM) for 24h were subjected to CHX pulse-chase assay. Quantification of grayscale ratio of FLIP/GAPDH by Photoshop software were shown in the right-hand panel. (**E**) U87 cells pretreated with IPZ (16 μM) for 24h or expressing shKPNB1s were treated with Act D (5 μg/ml) and MG132 (25 μM) for 2h, then subjected to western blot. GAPDH was used as the loading control.

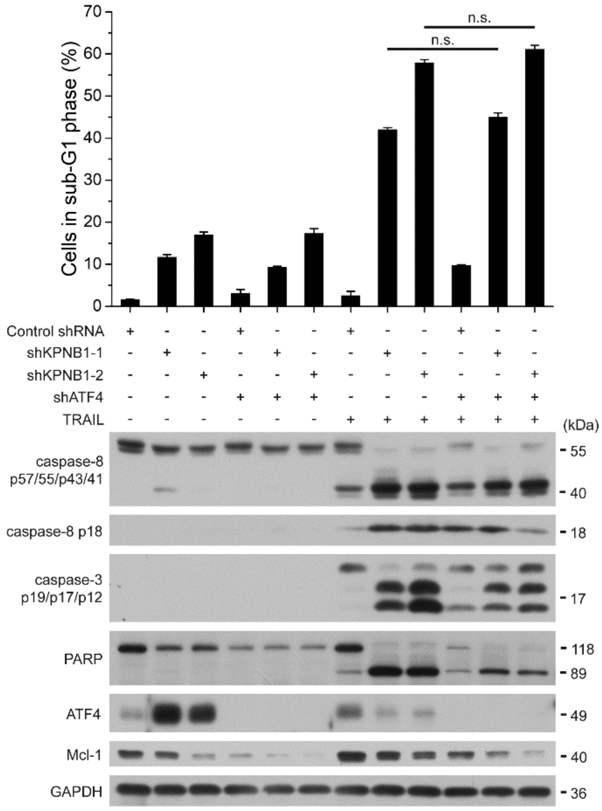

Supplementary Figure S6. (**A**) U87 cells expressing shKPNB1s and/or shATF4 were treated with TRAIL for 24h and subjected to flow cytometry and western blot. Results represent mean±SD from three independent experiments. n.s.: not significant. (**B**) U87 cells expressing shATF4 were treated with TRAIL and subjected to western blot. GAPDH was used as the loading control.

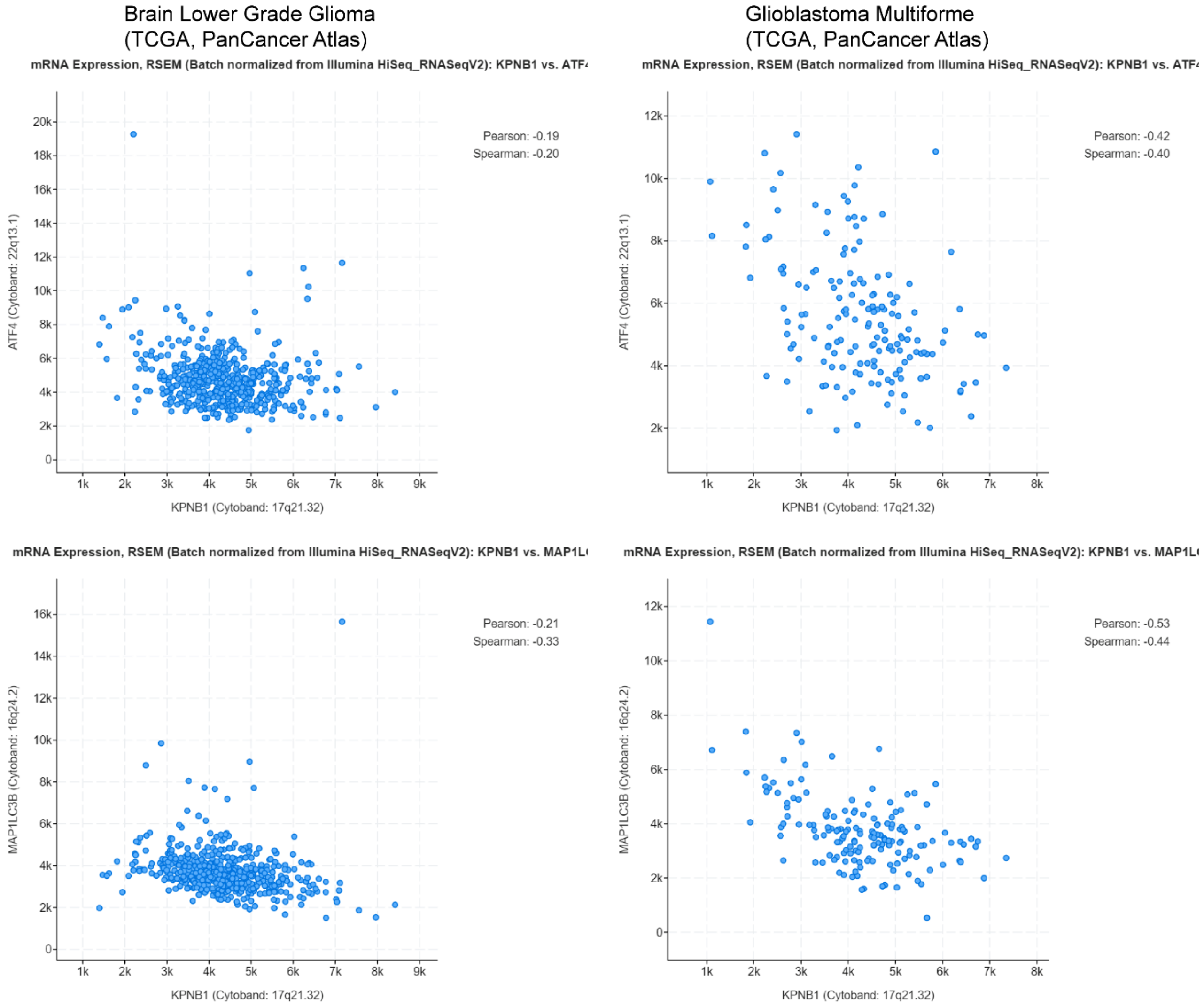

Supplementary Figure S7. Bioinformatics analysis of co-expression of KPNB1 and ATF4 (upper panel) or MAP1LC3B (lower panel) in brain lower grade glioma (left panel) and glioblastoma multiforme (right panel) based on TCGA PanCancer Atlas using the cBioPortal database.

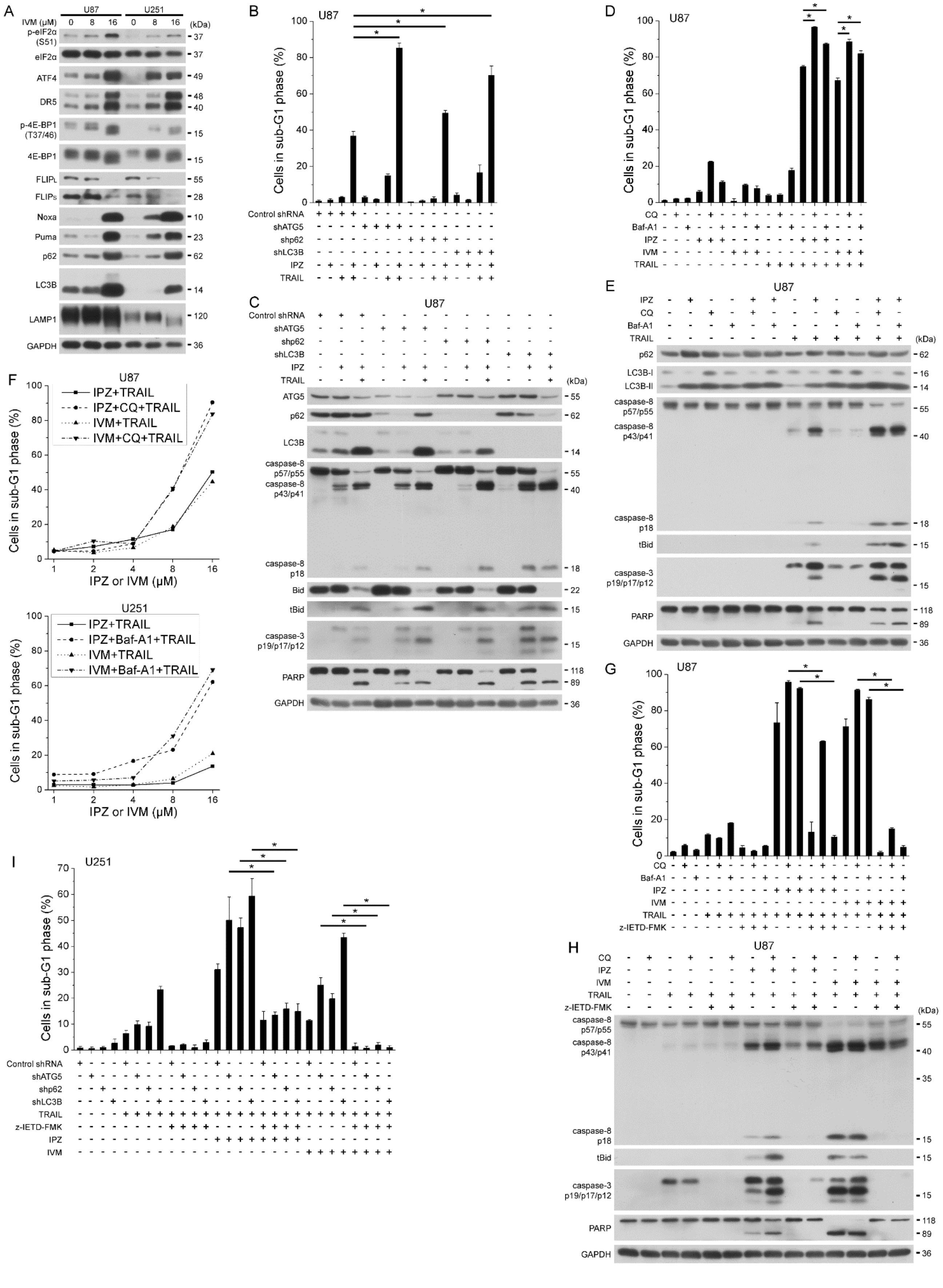

Supplementary Figure S8. (**A**) U87 and U251 cells were treated with indicated concentration of IVM for 24h and subjected to western blot. (**B**) and (**C**) U87 cells expressing either shATG5, shp62 or shLC3B were treated with IPZ (16 μM) for 24h and further with TRAIL and subjected to flow cytometry **B** and western blot **C**. (**D**) and (**E**) U87 cells were

treated with CQ (40 μM) or Baf-A1 (5 nM) along with IPZ (16 μM) or IVM (16 μM) for 24h and further with TRAIL, then subjected to flow cytometry **D** and western blot **E**. (**F**) U87 and U251 cells were treated with indicated concentration of IPZ or IVM for 24h and further with TRAIL (U87, 30 ng/ml; U251, 100 ng/ml) for 24h, then subjected to flow cytometry. (**G**) and (**H**) U87 cells were treated with CQ (40 μM) or Baf-A1 (5 nM) along with IPZ (16 μM) or IVM (16 μM) for 24h and further with z-IETD-FMK (20 μM) and TRAIL for 24h, then subjected to flow cytometry **G** and western blot **H**. (**I**) U251 cells expressing shATG5, shp62 or shLC3B were treated with IPZ (16 μM) or IVM (16 μM) for 24h and further with z-IETD-FMK (20 μM) and TRAIL (100 ng/ml) for 24h, then subjected to flow cytometry. GAPDH was used as the loading control. Flow cytometry results represent mean±SD from three independent experiments. *$P < 0.05$.

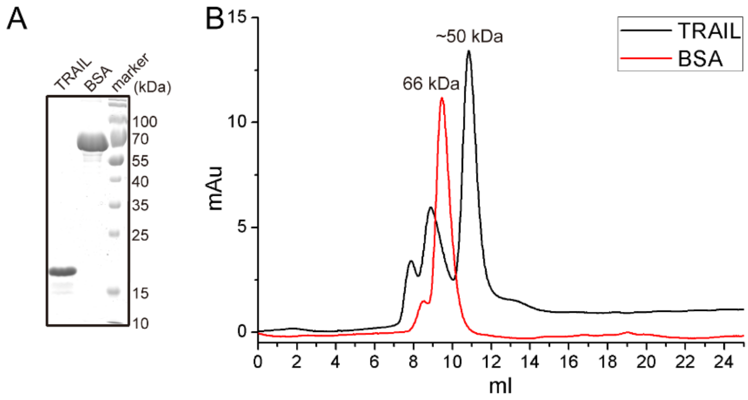

Supplementary Figure S9. (**A**) Purified recombinant soluble TRAIL and BSA standard (0.4 mg/ml) were analyzed by SDS-PAGE and stained with Coomassie Blue. (**B**) The oligomeric state of TRAIL was analyzed by gel filtration chromatography. Samples were run at 0.4 ml/min velocity. View from the comparison of TRAIL and BSA, TRAIL showed one peak corresponding to ~50 kDa.

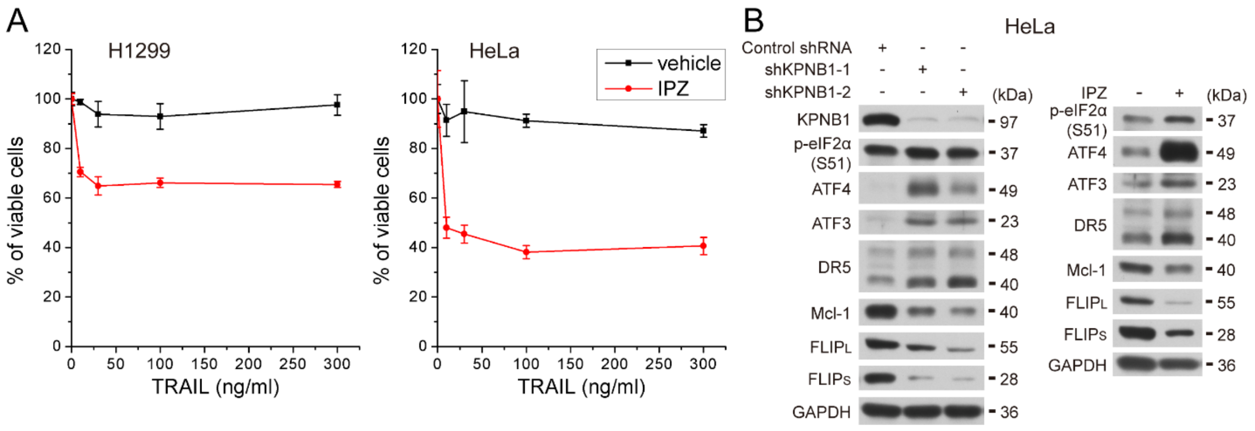

Supplementary Figure S10. (**A**) Non-small cell lung cancer cell line H1299 and cervical cancer cell line HeLa cells were treated with IPZ (16 µM) for 24h and further with indicated concentration of TRAIL for 24h. Cell viability was measured by MTT assay. Results represent the mean±SD from one of the three independent experiments in triplicates. (**B**) Western blot analysis of protein levels in HeLa cells either expressing shKPNB1s or treated with IPZ (16 µM) for 24h. GAPDH was used as the loading control.
